# Pre-operative aerobic exercise on metabolic health and surgical outcomes in patients receiving bariatric surgery: A pilot trial

**DOI:** 10.1371/journal.pone.0239130

**Published:** 2020-10-02

**Authors:** Nicole M. Gilbertson, Julian M. Gaitán, Victoria Osinski, Elizabeth A. Rexrode, James C. Garmey, J. Hunter Mehaffey, Taryn E. Hassinger, Sibylle Kranz, Coleen A. McNamara, Arthur Weltman, Peter T. Hallowell, Steven K. Malin

**Affiliations:** 1 Department of Kinesiology, University of Virginia, Charlottesville, Virginia, United States of America; 2 Robert M. Berne Cardiovascular Research Center, University of Virginia, Charlottesville, Virginia, United States of America; 3 Department of Surgery, University of Virginia, Charlottesville, Virginia, United States of America; 4 Department of Medicine, University of Virginia, Charlottesville, Virginia, United States of America; Universita degli Studi di Roma La Sapienza, ITALY

## Abstract

**Objective:**

Examine if adding aerobic exercise to standard medical care (EX+SC) prior to bariatric surgery improves metabolic health in relation to surgical outcomes.

**Methods:**

Fourteen bariatric patients (age: 42.3±2.5y, BMI: 45.1±2.5 kg/m^2^) met inclusion criteria and were match-paired to pre-operative SC (n = 7) or EX+SC (n = 7; walking 30min/d, 5d/wk, 65–85% HR_peak_) for 30d. A 120min mixed meal tolerance test was performed pre- and post-intervention (~2d prior to surgery) to assess insulin sensitivity (Matsuda Index) and metabolic flexibility (indirect calorimetry). Aerobic fitness (VO_2_peak), body composition (BodPod), and adipokines (adiponectin, leptin) were also measured. Omental adipose tissue was collected during surgery to quantify gene expression of adiponectin and leptin, and operating time and length of hospital stay were recorded. ANOVA and Cohen’s d effect size (ES) was used to test group differences.

**Results:**

SC tended to increase percent body fat (*P* = 0.06) after the intervention compared to EX+SC. Although SC and EX+SC tended to raise insulin sensitivity (*P* = 0.11), EX+SC enhanced metabolic flexibility (*P* = 0.01, ES = 1.55), reduced total adiponectin (*P* = 0.01, ES = 1.54) with no change in HMW adiponectin and decreased the length of hospital stay (*P* = 0.05) compared to SC. Albeit not statistically significant, EX+SC increased VO_2_peak 2.9% compared to a 5.9% decrease with SC (*P* = 0.24, ES = 0.91). This increased fitness correlated to shorter operating time (r = -0.57, *P* = 0.03) and length of stay (r = -0.58, *P* = 0.03). Less omental total adiponectin (r = 0.52, *P* = 0.09) and leptin (r = 0.58, *P* = 0.05) expression correlated with shorter operating time, and low leptin expression was linked to shorter length of stay (r = 0.70, *P* = 0.01), and low leptin expression was linked to shorter length of stay (r = 0.70, *P* = 0.01).

**Conclusion:**

Adding pre-operative aerobic exercise to standard care may improve surgical outcomes through a fitness and adipose tissue derived mechanism.

## Introduction

Bariatric surgery is an effective treatment for reducing obesity [1, 2] and related co-morbidities such as adipose tissue derived inflammation [1–6] and insulin insensitivity [1–6]. The number of intra- and post-operative complications and length of operating time as well as length of hospital stay though varies among patients [7–9]. The exact reason for this large inter-subject variability to bariatric surgery outcomes is unclear, but decreased inflammation, elevated insulin sensitivity, and greater fitness at the time of surgery is linked to improved surgical outcomes [10]. Thus, enhancing metabolic health prior to surgery may improve patient outcomes.

Low calorie diets (LCD) are standard medical practice prior to bariatric surgery and typically consist of meal replacement shakes for breakfast and lunch along with sensible snacks and a lean protein, high fiber dinner [11–13]. These 2–4 week pre-operative LCD reduce liver size prior to surgery and often coincide with lower surgical complication rates and improved surgical outcomes [11–15]. Cardiorespiratory fitness also impacts surgery outcomes, as a peak oxygen uptake (VO_2_peak) greater than 15.8 ml/kg/min at the time of bariatric surgery is related to shorter operating time and fewer surgical complications [16]. The latter is supported by a prospective pre-operative exercise training study that showed that exercise prior to surgery induces greater weight loss and quality of life [17, 18] compared to standard medical care (SC). Through a series of recent studies, we evaluated the effect of a 2-week LCD (about 1200kcal/d) based on pre-operative standard medical care nutrition recommendations for adults undergoing bariatric surgery, with or without an energy deficit matched aerobic exercise intervention. Results collectively indicate that LCD+exercise increased aerobic fitness, maintained post-prandial carbohydrate (CHO) utilization, and improved glucose tolerance when compared with LCD alone [19, 20]. These findings suggest that exercise may augment the ability of a LCD to enhance insulin-regulated glycemia and metabolic health. Whether adding aerobic exercise to pre-operative SC prior to bariatric surgery improves metabolic health or surgical outcomes at the time of surgery remains unknown. The purpose of the present pilot study was to close this knowledge gap by examining if adding exercise to SC (EX+SC) improved metabolic health and surgical outcomes compared to SC alone in patients receiving bariatric surgery. We hypothesized that pre-operative EX+SC would increase cardiorespiratory fitness and insulin sensitivity and decrease adipose-derived inflammation compared to SC. Further, we anticipated that these changes in metabolic health would correlate with improved bariatric surgery outcomes.

## Methods

### Subjects

**Fig 1:**
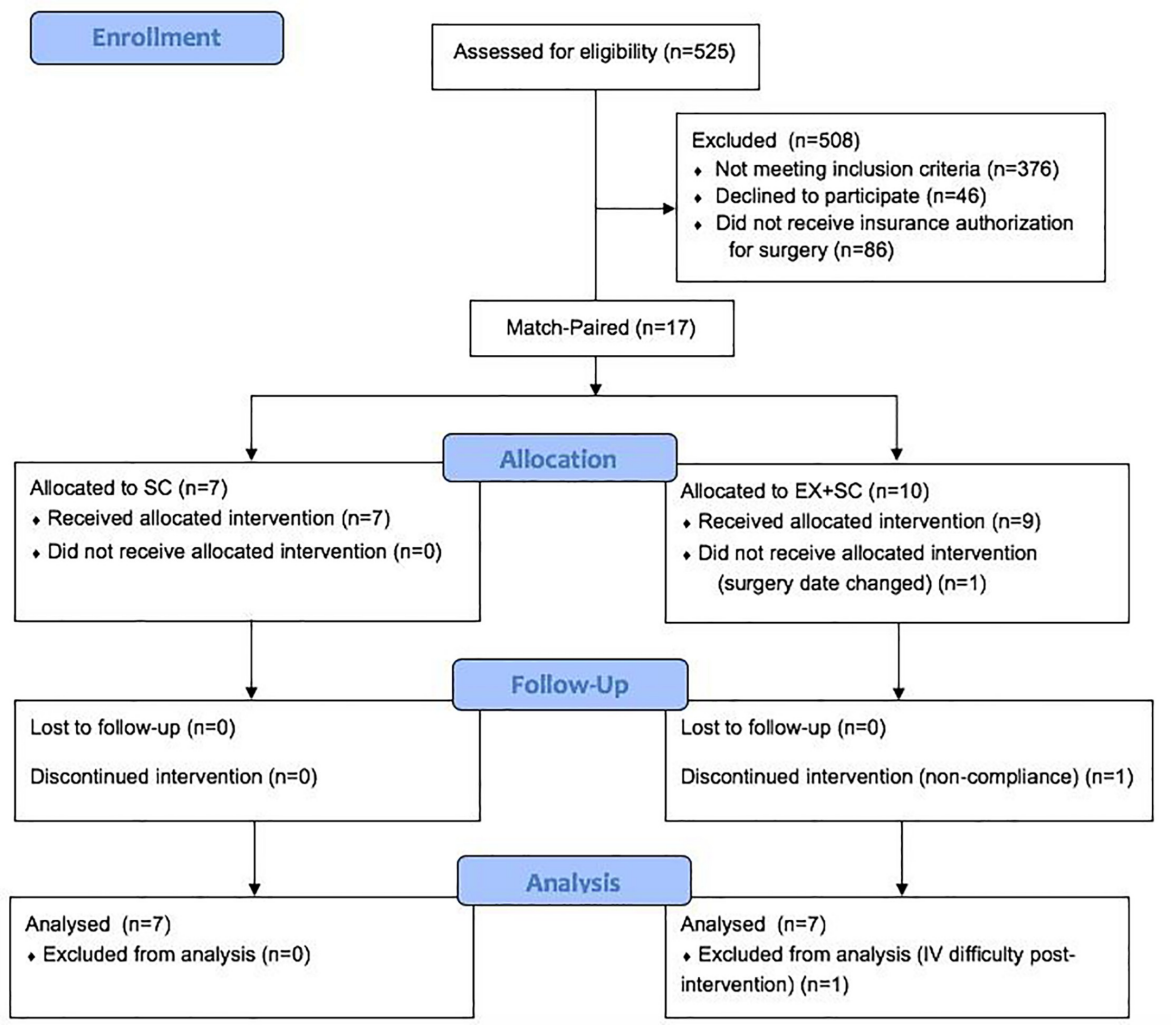
CONSORT flow diagram.

Bariatric surgery candidates were recruited from the University of Virginia surgical clinic between 2015–2018 (Fig 1). Adults aged 18–70 years were included for participation in this study if they were undergoing their first Roux-en-Y gastric bypass (RYGB) or sleeve gastrectomy (SG) procedure, sedentary (<60 min/wk of physical activity as determined by a pre-screening questionnaire), and obese (BMI of 30–70 kg/m^2^). Participants were excluded if they were pregnant or breastfeeding, taking medications known to alter body weight, diagnosed with insulin dependent diabetes, had a history of cardiovascular disease, or diagnosed with cancer in the past 5 years. P.T.H. completed all physical examinations and medically cleared subjects to participate in the study. Participants were then match-paired based on BMI, sex, and surgery type to EX+SC or SC (Table 1). All outcomes were assessed pre- and post-intervention (i.e. after 30 days of intervention and ~2 days prior to surgery). This study was approved by the University of Virginia’s Institutional Review Board. All participants provided written and verbal informed consent. This trial was registered at clinicaltrials.gov (# NCT03854981).

**Table 1:** Effect of SC and EX+SC on subject characteristics.

	SC		EX+SC		ANOVA (*P*-value)			Effect Size
	PRE	POST	PRE	POST	G	T	GxT	Cohen’s *d*
*Demographics*								
Age (years)	39.0±5.3		45.6±4.8					
Sex	1 M, 6 F		7 F					
Surgical Procedure	3 RYGB, 4 SG		3 RYGB, 4 SG					
Race	5 C, 2 AA		5 C, 1 AA, 1 PI					
*Body Composition*								
Weight (kg)	128.4±10.9	127.8±10.3	116.5±11.9	116.0±11.8	0.47	0.53	0.96	0.03
BMI (kg/m^2^)	46.4±3.0	46.2±2.7	43.9±4.2	43.7±4.1	0.63	0.50	0.93	0.05
FFM (kg)	60.3±3.3	58.8±3.2	54.7±5.1	54.1±4.4	0.39	0.05	0.39	0.48
Fat Mass (kg)	67.1±8.3	68.0±7.8	61.2±7.6	60.9±8.0	0.57	0.66	0.36	0.52
% Body Fat	51.7±2.4	52.8±2.4	52.3±1.4	52.2±1.8	0.99	0.15	0.06	1.10
*Fitness*								
VO_2_peak (L/min)	2.51±0.14	2.36±0.13	2.33±0.17	2.31±0.11	0.51	0.35	0.46	0.42
VO_2_peak (mL/kg/min)	20.4±1.5	19.2±1.6	20.5±1.3	21.1±2.0	0.66	0.65	0.24	0.91
VO_2_peak (mL/kg-FFM/min)	42.4±3.0	40.9±3.1	43.4±2.8	44.0±3.5	0.64	0.77	0.46	0.41
*Energy Availability*								
Caloric Intake (kcal/d)	1922±273	1827±414	1987±232	1240±286	0.51	0.06	0.14	0.86
Carbohydrate (kcal/d) ||	732±119	811±220	912±122	478±134	0.50	0.05	0.04	1.06
Protein (kcal/d) ||	448±99	449±151	328±42	274±47	0.34	0.12	0.51	0.35
Fat (kcal/d)	746±96	561±94	790±87	446±132	0.79	0.003	0.28	0.61
RMR (kcal/kgBW/d)	12.0±0.4	12.7±0.7	11.0±0.6	11.4±1.1	0.16	0.55	0.87	0.10
RMR (kcal/kgFFM/d)	24.7±1.3	26.0±1.8	23.4±1.5	23.8±1.9	0.58	0.58	0.77	0.16

Data are means ± SEM. There was no difference in age between groups (*P* = 0.37), and there were no baseline differences between groups for any variable. ||Non-normally distributed data are presented in raw version for ease of interpretation. Abbreviations: Male (M); female (F); roux-en-y gastric bypass (RYGB); sleeve gastrectomy (SG); Caucasian (C); African American (AA); Pacific Island (PI); body mass index (BMI); fat free mass (FFM); group (G; SC vs. EX+SC); time (T; PRE vs. POST-INTERVENTION); group x time interaction (G x T).

### Body composition and cardiorespiratory fitness

Following an approximate 4 hour fast, body weight was measured to the nearest 0.01 kg on a digital scale with minimal clothing and without shoes and height was measured with a stadiometer to assess BMI. Fat mass and fat-free mass (FFM) were measured using air displacement plethysmography (BodPod, Concord, CA). VO_2_peak was determined using a treadmill exercise test with indirect calorimetry (Carefusion, Vmax CART, Yorba Linda, CA). Subjects self-selected a speed and grade was increased 2.5% every 2 minutes until volitional exhaustion. Criteria used to deem the test of peak level included 2 of the 3: respiratory exchange ratio (RER) >1.1, heart rate within 10 beats per minute of age predicted max, and/or rating of perceived exertion ≥ 17.

### Metabolic control

Subjects were instructed to avoid alcohol, caffeine, dietary supplements, and medication for 24 hours prior to testing. Participants completed a 3 day food diary prior to metabolic testing to identify nutrition intake and assess differences throughout study time points. Participants undergoing EX+SC completed exercise sessions 24 hours prior to metabolic testing and 18–48 hours prior to surgery. All participants were informed to avoid vigorous non-exercise physical activity approximately 24 hours prior to testing to isolate the effects of the exercise training on metabolic health and surgical outcomes.

### Mixed meal tolerance test (MMTT)

Subjects were admitted to the Clinical Research Unit at approximately 8:00 a.m. following an overnight fast. An intravenous catheter was placed in an antecubital vein, and fasting blood was collected to measure HMW adiponectin, total adiponectin, and leptin. We defined adiposopathy as the ratio of HMW adiponectin to leptin as previously reported [19]. Participants then consumed 4 fl. oz. of an Ensure Plus (Abbott Park, Illinois) shake (CHO 25g, fat 5.5g, protein 6.5g). Circulating glucose, free fatty acids (FFA), and insulin were determined at fasting and every 30 minutes up to 120 minutes after consumption of the mixed meal to assess total area under the curve (tAUC) and whole-body insulin sensitivity [21]. Indirect calorimetry (Carefusion, Vmax CART, Yorba Linda, CA) with a ventilated hood was utilized to assess resting metabolic rate as well as RER at 0, 60, and 120 minutes of the MMTT. Metabolic flexibility was determined by subtracting fasting RER from the average of post-prandial RER as previously reported [22].

### Surgical outcomes

All RYGB or SG surgical procedures were completed by one investigator (P.T.H.), who was blinded to participants intervention allocation. Operating time was defined as time of incision to close. Length of hospital stay was defined as the time of hospital admission to discharge. Surgical residents were responsible for hospital discharge and had no knowledge of the patient’s participation in the present study. Intra- and post-surgical complications were recorded (i.e. surgical site infection, unplanned intubation, pulmonary embolism, cardiovascular event, sepsis, etc.), and any visit to an emergency department or hospital re-admission within 30 days from discharge was noted.

### Adipose biopsy

At the time of surgery, an omental adipose tissue sample was obtained and immediately placed in a solution of phosphate buffer saline containing 0.1% glucose and 0.05mg/mL gentamycin. Omental adipose tissue was digested to isolate adipocytes using published methods [23]. Samples were then flash frozen and stored at -80°C until ribonucleic acid (RNA) extraction. Real-time polymerase chain reaction (PCR) was completed to quantify the gene expression of adiponectin and leptin in adipose tissue.

### Standard medical care (SC)

Participants met with registered dieticians, attended an education session with a registered nurse, and were cleared for bariatric surgery by a psychologist and the surgical team prior to study enrollment. Patients attended a pre-operative visit approximately 40 days prior to bariatric surgery. For 2 weeks prior to surgery, patients were instructed by registered dieticians to consume a meal replacement shake for breakfast and lunch, snacks of raw vegetables, a dinner composed of 4 oz. of lean protein and steamed vegetables, and sugar-free beverages.

### Exercise (EX)

Subjects participating in EX+SC also completed at home walking for 30 min/day, 5 days/week, at 65–85% heart rate peak (HR_peak_) for 30 days. Exercise adherence was monitored by the research team using an exercise diary and a A300 Polar fitness and activity tracker (Kempele, Finland). The Polar fitness monitor tracked the duration, heart rate, and calories expended per exercise session. The research team also conducted weekly check-ins by texting, emails, and/or phone calls with study participants to promote adherence.

### Biochemical and adipose tissue analysis

Plasma glucose samples were analyzed immediately using a YSI 2300 StatPlus Glucose Analyzer system (Yellow Springs, OH). All other samples were collected in EDTA tubes, centrifuged for 10 minutes at 4°C and 3000 rpm, and stored at -80°C until later analysis. Samples were batch analyzed in duplicate to minimize inter-assay variability. Circulating HMW adiponectin, total adiponectin, and insulin (Alpco, Salem, NH) as well as leptin (Millipore, Billerica, MA) was determined using enzyme-linked immunosorbent assay kits. FFAs were analyzed using enzymatic colorimetric assay (Wako Diagnostics, Richmond, VA). RNA was extracted from adipocytes using Trizol extraction. One μg of omental RNA was then treated with deoxyribosnuclease (Invitrogen, Carlsbad, CA) and used to reverse transcribe complementary deoxyribonucleic acid (cDNA) using an iScript cDNA synthesis kit (BioRad, Hercules, CA). To quantify gene expression, cDNA was combined with Taqman primer probes (HPRT1-VIC plus one additional FAM-conjugated primer-probe) and SensiMix™ II Probe Kit (BioLine, London, UK). Semi-quantitative real-time PCR was performed on a CFX96 Real-Time System with an annealing temperature of 60°C for all reactions (BioRad, Hercules, CA). Data were calculated by the ΔΔCt method and expressed in arbitrary units that were normalized to HPRT1 levels.

### Statistical analysis

Power was calculated based on our primary outcome insulin sensitivity. It was determined that 5 obese adults would be needed to show the effect of short-term EX on insulin sensitivity (delta of 0.5, SD of 0.9 with 80% power and alpha of 0.05) [24]. Overall, 17 adults met eligibility criteria and were match-paired to SC (n = 7) or EX+SC (n = 10) (Fig 1). Three subjects were excluded from analyses due to non-compliance to the exercise intervention (n = 1 EX+SC), failure to obtain blood post-intervention (n = 1 EX+SC, e.g. IV difficulty), and inability to complete intervention prior to surgery (n = 1 EX+SC). Therefore, 14 adults (n = 7 SC, n = 7 EX+SC) completed the study and were included in analysis, but omental adipose tissue was obtained and analyzed in 12 subjects (n = 5 SC, n = 7 EX+SC). Data were analyzed by using SPSS Version 26 (IBM Analytics, Armonk, NY). Outliers were determined by being >2 standard deviations from the mean, and outliers were removed for analysis of insulin tAUC_120min_ (n = 1 SC), 120 minute FFA (n = 1 EX+SC), and resting metabolic rate (kcal/kg body weight/day; n = 1 SC). Normality was tested using Shapiro-Wilk test of normality, and non-normally distributed data, including leptin, total adiponectin, gene expression of adiponectin and leptin in adipose tissue, 120 minute insulin, protein intake, and carbohydrate intake, were log-transformed for analysis. Baseline differences, length of operating time and hospital stay, as well as omental gene expression were analyzed using independent samples t-tests. The difference in the number of emergency department readmissions within 30 days of surgery between groups was analyzed using Fisher Exact Test. All other variables were analyzed using a 2x2 repeated measure analysis of variance. Cohen’s d effect sizes (ES) were also calculated on the interaction of treatments, and physiological relevance was interpreted as small (d = 0.2), medium (d = 0.5), or large (d = 0.8). Pearson’s correlation was used to assess associations. Significance was set at *P*≤0.05, and trends were accepted at 0.05 ≤ x ≤ 0.10. Data are presented as mean ± SEM.

## Results

### Diet and exercise characteristics

EX+SC participants completed 18.4±3.0 exercise sessions for 37.3±2.6 min/session at 75.7±0.02% of their HR_max_ which equated to 248.8±24.0 kcal/session. Total calorie (*P* = 0.06) and fat (*P* = 0.003) intake decreased during the interventions (Table 1), and EX+SC had a greater effect than SC for reductions in calorie (ES = 0.86) and fat intake (ES = 0.61). EX+SC decreased intake of CHO (*P* = 0.04; ES = 1.06) compared to increased intake with SC (Table 1).

### Body composition and fitness

SC and EX+SC elicited no changes in body weight or BMI over the study, however, EX+SC had a medium effect size (ES = 0.52) for decreased fat mass compared to an increase with SC. There was a reduction in FFM (Table 1*; P* = 0.05) with both interventions, but SC had a greater reduction than EX+SC (ES = 0.48). SC tended to increase while EX+SC maintained (Table 1*; P* = 0.06, ES = 1.10) percent body fat over the study. EX+SC had a large effect size (ES = 0.91) for increased VO_2_peak (mL/kg/min) compared to SC.

### Blood substrates

**Fig 2:**
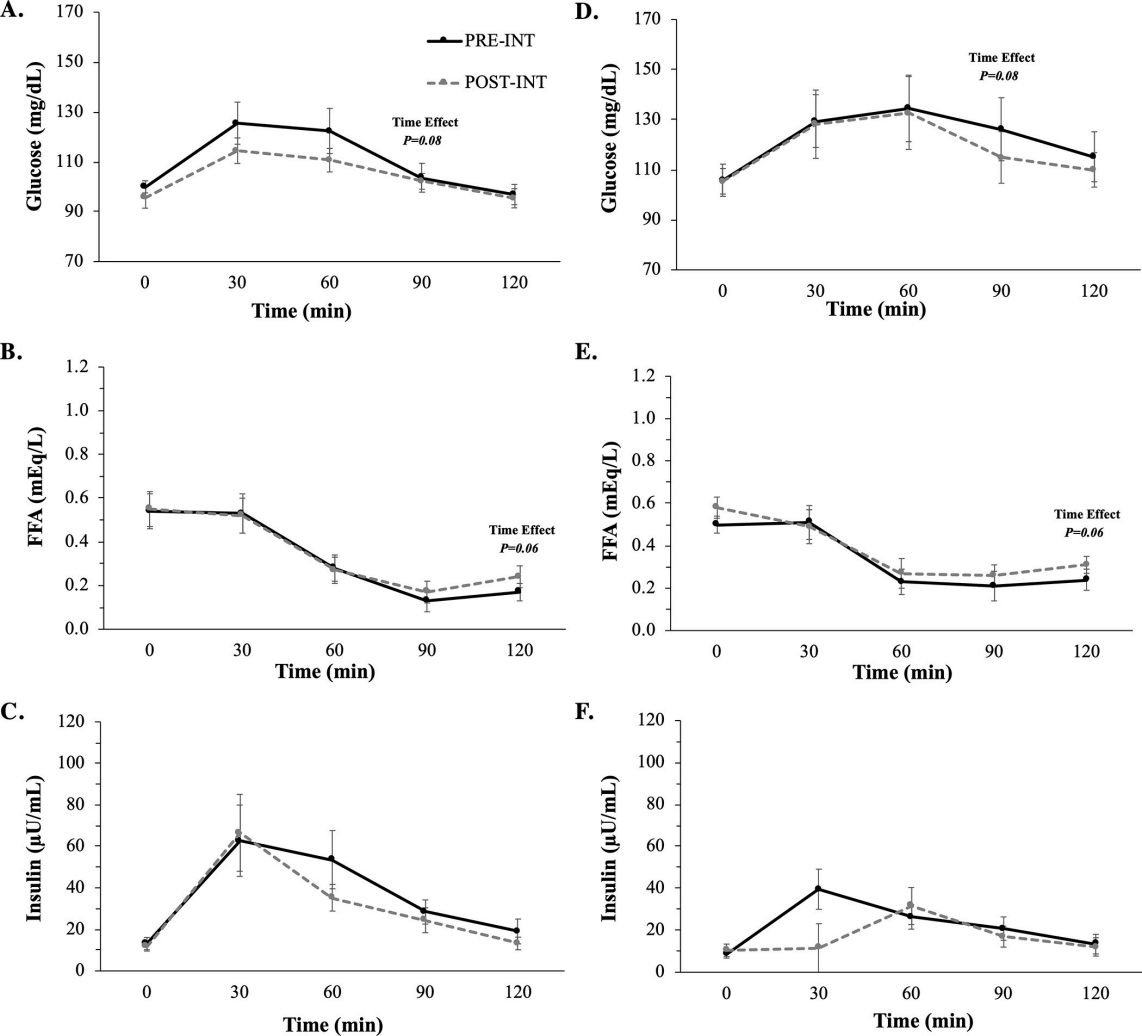
The effect of SC (*A-C*) and EX+SC (*D-F*) on mixed meal tolerance test glucose, free fatty acid (FFA), and insulin curves. Data are means ± SEM. There was a trend for a time effect for 90 minute glucose (*P* = 0.08) and 120 minute FFA (*P* = 0.06). Conversions: Glucose, 1.00 mmol/L = 18.01 mg/dL; FFA 1.00 mEq/L = 1.00 mmol/L; Insulin, 1.00 μU/mL = 6.95 pmol/L.

Fasting (Fig 2) substrates as well as glucose, FFA, and insulin tAUC_120min_ (Table 2**)** were unaltered with SC or EX+SC.

**Table 2:** Effect of SC and EX+SC on metabolic outcomes and adipokines.

	SC		EX+SC		ANOVA (*P*-value)			Effect Size
	PRE	POST	PRE	POST	G	T	GxT	Cohen’s *d*
Insulin Sensitivity	4.99±0.72	6.36±1.34	8.49±2.15	10.71±3.41	0.20	0.11	0.69	0.33
Glucose tAUC (mg/dL•120min)	13497±739	12687±424	15015±1277	14501±1160	0.22	0.15	0.74	0.18
(mg/dL•120min)in								
FFA tAUC (mmol/L•120min)	39±7	41±7	40±7	44±6	0.81	0.61	0.82	0.13
(mEq/L•120min)								
Insulin tAUC (μU/mL•120min)	3859±541	3736±961	2932±562	3093±717	0.38	0.97	0.79	0.14
(μU/ml•120min) ||								
*Adipokines* (ng/mL)								
Leptin ||	100.4±17.2	105.0±20.9	90.9±13.5	87.8±18.5	0.63	0.67	0.55	0.33
HMW Adiponectin	1112.9±226.9	1179.6±229.5	2375.0±608.2	2240.0±520.8	0.98	0.05	0.98	0.59
Total Adiponectin ||	4877.1±629.3	4972.9±696.1	6216.4±1252.2	5360.6±1062.8	0.83	0.04	0.01	1.54
HMW:Total	0.22±0.02	0.23±0.02	0.39±0.06	0.44±0.07	0.05	0.20	0.09	0.68
HMW:Leptin	13.6±3.6	14.6±3.7	29.3±8.7	30.1±7.6	0.10	0.62	0.98	0.04

Data are means ± SEM. HMW adiponectin and the ratio of HMW to total adiponectin were covaried for baseline HMW adiponectin group differences (*P* = 0.09). ||Non-normally distributed data are presented in raw version for ease of interpretation. Conversions: Glucose, 1.00 mmol/L = 18.01 mg/dL; FFA, 1.00 mmol/L = 1.00 mEq/L; Insulin, 1.00 μU/mL = 6.95 pmol/L; Adipokines, 1.00 ng/mL = 0.001 μg/mL. Abbreviations: Total area under the curve (tAUC) for 120 min; free fatty acids (FFA); high molecular weight (HMW); group (G; SC vs. EX+SC); time (T; PRE vs. POST); group x time interaction (G x T).

### Adipokines

EX+SC had a reduction in total adiponectin compared to a slight increase with SC (Table 2; *P* = 0.01, ES = 1.54). SC had a medium effect size (Table 2; ES = 0.59) for increased HMW adiponectin compared to a decrease with EX+SC. EX+SC tended to increase the ratio of HMW to total adiponectin compared to SC (Table 2; *P* = 0.09, ES = 0.68), and there was a significant group effect (*P* = 0.05). EX+SC had a small effect size for decreased circulating leptin (Table 2; ES = 0.33), but the ratio of HMW adiponectin to leptin did not change over the study (Table 2). There was no difference between treatments in omental gene expression of adiponectin (SC 1.29±0.29 vs. EX+SC 1.92±0.67a.u.; *P* = 0.65) or leptin (SC 1.50±0.39 vs. EX+SC 1.21±0.46a.u.; *P* = 0.47).

### Insulin sensitivity and fuel use

**Fig 3:**
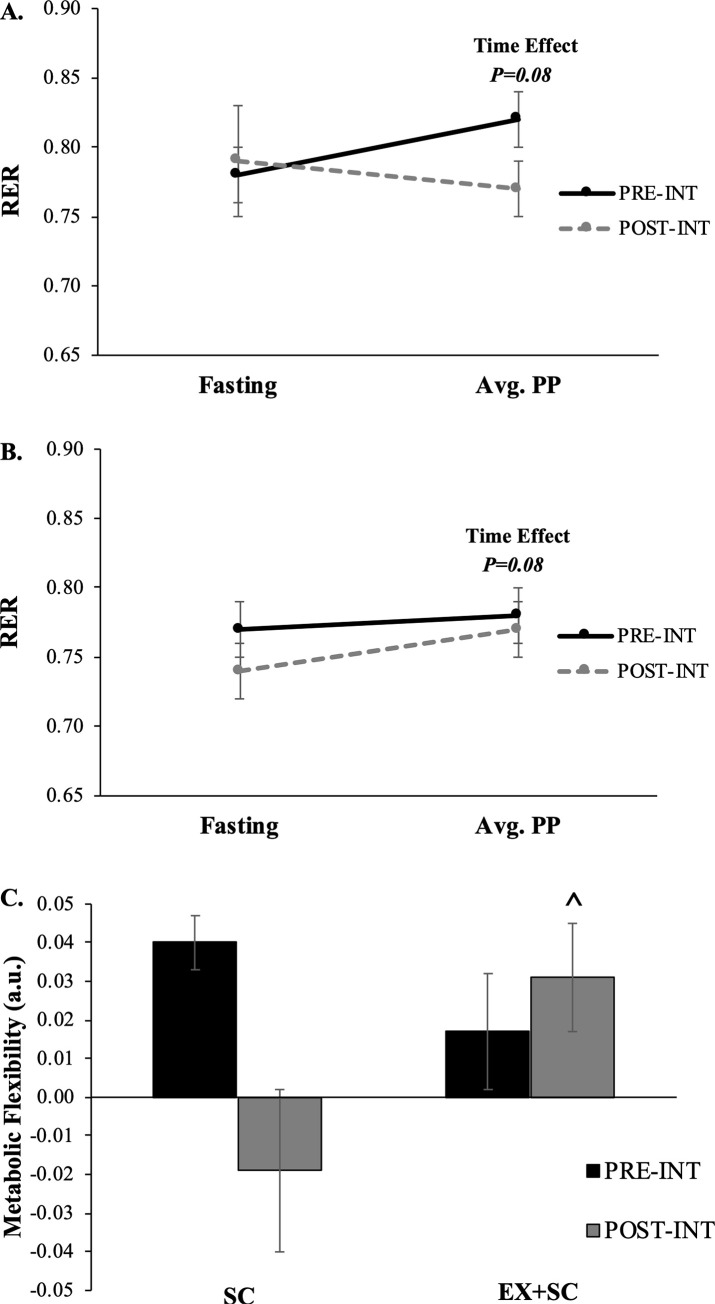
The effect of SC (*A*) and EX+SC (*B*) on fasting and average post-prandial respiratory exchange ratio as well as metabolic flexibility (*C*). Data are means ± SEM. There is a trend (*P* = 0.08) for a time effect for average post-prandial RER. ^Denotes a significant (*P* = 0.01) group x time interaction. Metabolic flexibility was determined by subtracting fasting respiratory exchange ratio (RER) from the average of post-prandial (avg. PP) RER.

Insulin sensitivity tended to increase over the study (Table 2; *P* = 0.11) for SC and EX+SC, however, EX+SC had a small effect size for a greater increase in insulin sensitivity than SC (ES = 0.33). Resting metabolic rate was not altered over the course of the study with either treatment (Table 1; *P*≥0.55). The average post-prandial (Fig 3; *P* = 0.08) RER tended to decrease comparably with both treatments, although EX+SC increased metabolic flexibility compared to a lowered effect with SC (Fig 3; *P* = 0.01, ES = 1.55).

### Surgical outcomes

EX+SC decreased length of hospital stay compared to SC (2479.1±264.1 vs. 3404.6±344.4 mins; *P* = 0.05). There was no difference between treatments in length of surgery (SC 128.0±18.9 vs. EX+SC 126.7±15.3 mins; *P* = 0.96). No intra- or post-surgical outcomes were noted, and no participants were readmitted to the hospital within 30 days of surgery. Three participants (SC n = 2, EX+SC n = 1) visited the emergency department within 30 days of surgery.

### Correlations

**Fig 4:**
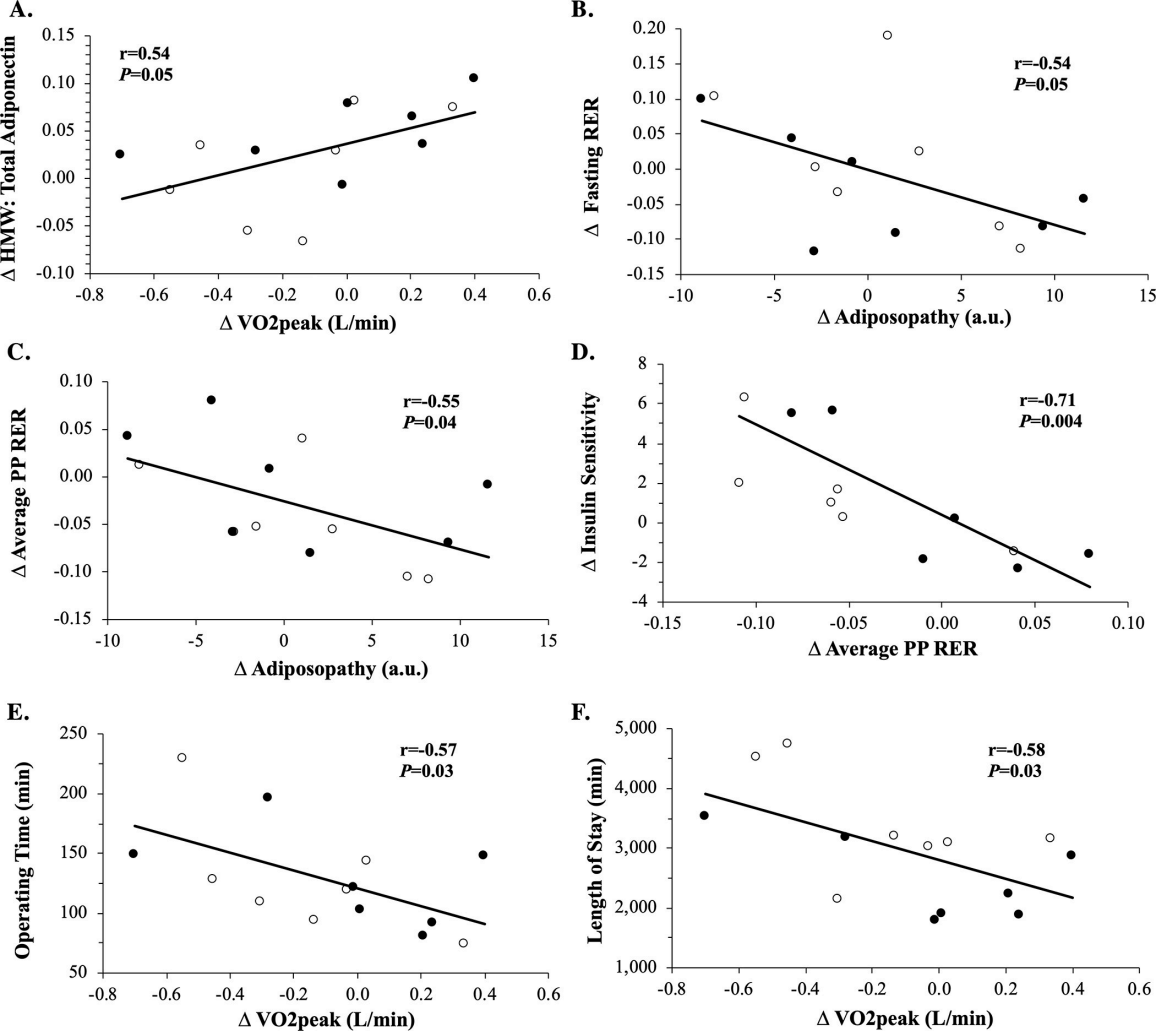
Correlations in metabolic outcomes following the intervention. The change (Δ) in VO_2_peak (L/min) to the Δ in the ratio of high molecular weight (HMW) to total adiponectin (*A*). The Δ in HMW adiponectin:leptin to the Δ in fasting respiratory exchange ratio (RER) (*B*) and Δ in average post-prandial (PP) RER (*C*). The Δ in peripheral insulin sensitivity to the Δ in PP RER (*D*). The Δ in VO_2_peak (L/min) to operating time (*E*) and length of hospital stay (*F*).

Increased absolute VO_2_peak (L/min) was associated with increased lean mass (r = 0.59, *P* = 0.03) and ratio of HMW to total adiponectin (Fig 4A; r = 0.54, *P* = 0.05) as well as reduced length of surgery (Fig 4E; r = -0.57, *P* = 0.03) and hospital stay (Fig 4F; r = -0.58, *P* = 0.03). Similarly, increased relative VO_2_peak (mL/kg/min) was associated with increased lean mass (r = 0.53, *P* = 0.05) and ratio of HMW to total adiponectin (r = 0.68, *P* = 0.008) as well as reduced hospital stay (r = -0.52, *P* = 0.06) but not length of surgery (r = -0.43, *P* = 0.13). Lower body weight post-intervention correlated with less omental total adiponectin (r = 0.69, *P* = 0.01) and leptin (r = 0.81, *P* = 0.002) expression at the time of surgery. Higher absolute VO_2_peak post-intervention was associated with less omental total adiponectin (r = -0.67, *P* = 0.02) and leptin (r = -0.81, *P* = 0.002) expression at the time of surgery. Less omental total adiponectin (r = 0.52, *P* = 0.09) and leptin (r = 0.58, *P* = 0.05) expression correlated with shorter operating time, and low leptin expression was linked to shorter length of stay (r = 0.70, *P* = 0.01). However, changes in fat mass did not correlate with either shorter operating time (r = -0.26, *P* = 0.35) or length of stay (r = -0.06, *P* = 0.82). Increased whole-body insulin sensitivity was associated with decreased average post-prandial RER (Fig 4D; r = -0.71, *P* = 0.004). The increased ratio of HMW adiponectin to leptin correlated to a decreased fasting (Fig 4B; r = -0.54, *P* = 0.05) and average post-prandial (Fig 4C; r = -0.55, *P* = 0.04) RER. Lowered fasting RER was linked to fat loss (r = 0.55, *P* = 0.04), and this decreased fat mass correlated with an elevated ratio of HMW to total adiponectin (r = -0.54, *P* = 0.05). Decreased caloric intake was associated with decreased leptin (r = 0.62, *P* = 0.02) and increased metabolic flexibility (r = -0.60, *P* = 0.03). Reduced carbohydrate intake also correlated with lowered leptin (r = 0.63, *P* = 0.02) and elevated metabolic flexibility (r = -0.63, *P* = 0.02).

## Discussion

The major finding of the present pilot study is that patients undergoing pre-operative EX+SC prior to bariatric surgery had a shorter length of hospital stay than patients undergoing pre-operative SC. This finding is clinically relevant as reducing length of hospital stay is directly linked to decreased risk for infection and health care cost [25–27]. A LCD is often reported in the literature as standard medical practice prior to bariatric surgery to decrease operating time via pre-surgical weight loss and increased insulin sensitivity [11, 12, 14, 15]. Herein, there were no statistically significant differences in weight loss, insulin sensitivity, or operating time between EX+SC and SC. This suggests that exercise is unlikely to enhance the effects of SC on surgical outcomes via weight loss and/or insulin sensitivity per se. Interestingly, EX+SC had a large effect size for reductions in caloric and carbohydrate intake as well as a medium effect size for reduction in fat intake compared to SC. The reason that EX+SC had a greater effect for a reduction in dietary intake than SC are outside of the scope of the present study, but it might be due either a direct effect of exercise to impact appetite or through the inadvertent behavioral technique of prompting to foster exercise compliance [28, 29]. Regardless, it is possible that changes in dietary intake affected length of stay in those who exercised through reductions in body fat. Indeed, EX+SC was shown to have medium to large effect size effects for decreasing body fat. These findings in body fat are potentially pertinent since prior work shows that fat mass is independently associated with length of hospital stay in patients admitted to the hospital for medical or surgical reasons [30]. While further research is warranted to understand the impact of dietary changes on reducing length of stay, fat mass reduction is unlikely to be the sole factor for decreasing length of hospital stay in those that exercised though as we noted no correlation with surgical outcomes. In contrast, we identified a moderate to large effect size of EX+SC to increase VO_2_peak, and this aerobic fitness gain was associated with a decreased operating time (Fig 4E) and length of hospital stay (Fig 4F). Therefore, our findings suggest that raising aerobic fitness prior to bariatric surgery is important for reducing length of hospital stay.

There are several reasons why pre-operative exercise fitness gains could have contributed to reduced operating time and/or length of stay. Loss of skeletal muscle mass is a predictor of mortality following hepatic inter-arterial therapy and radical cystectomy for bladder cancer [31, 32], thereby suggesting muscle mass plays a role in surgical outcomes. Because caloric restriction can promote skeletal muscle mass loss due to protein degradation and decreased rates of protein synthesis [33], it would be expected that exercise counteracts these effects of diet [33, 34]. Indeed, our data suggest that pre-operative EX+SC resulted in a 1.1% reduction in fat free mass compared with a 2.5% decrease with SC prior to bariatric surgery. The reason muscle mass could contribute to improved length of stay post-surgery may specifically relate to the quality of skeletal muscle following exercise [35–37]. Our data suggests that preservation of fat free mass was directedly related to pre-operative EX+SC significantly increasing metabolic flexibility compared to SC (Fig 3), which is consistent with prior work in people with and without impaired glucose metabolism [22, 37–39]. This study was not designed to determine the mechanism by which exercise enhanced metabolic flexibility, but might relate to increased skeletal muscle oxidative capacity [35] and/or mitochondrial biogenesis [36]. Alternatively, we cannot exclude the possibility though that energy/carbohydrate availability via dietary changes impacted substrate oxidation [37–39], as increased metabolic flexibility in the present study was associated with a reduction in caloric and carbohydrate intake. In either case, the enhanced ability of EX+SC to oxidize nutrients at the time of surgery could potentially explain the shorter length of hospital stay, because the onset of surgical stress causes the body to mobilize nutrients to support tissue healing and protein synthesis. The flux of nutrients results in decreased hepatic and skeletal muscle insulin sensitivity [40, 41], which can last for >5 days [42]. Interestingly, the degree of post-operative insulin insensitivity is significantly associated with the length of hospital stay [41]. Future work should measure insulin sensitivity and metabolic flexibility at the time of surgery and immediately post-operatively to confirm these potentially beneficial effects of pre-operative EX+SC.

The increase in VO_2_peak may also be linked to a shorter operating time and length of stay through an adipocyte related mechanism. We showed that higher VO_2_peak post-intervention correlated to less omental gene expression of adiponectin and leptin at the time of surgery. Less omental adiponectin expression correlated with shorter operating time, and low leptin expression was linked to shorter length of stay. While less total adiponectin expression in relation to shorter operating time may appear counter intuitive since adiponectin is an anti-inflammatory hormone [43], it supports our findings that total circulating adiponectin was significantly reduced more with EX+SC than SC over the study. It is not possible to measure the omental gene expression of low, medium, or high molecular weight adiponectin with PCR, yet we report EX+SC significantly increased the ratio of HMW to total adiponectin compared to no change with SC. Further, we found that the pre-operative increases in the ratio of circulating HMW to total adiponectin was associated with enhanced VO_2_peak (Fig 4A). Maintaining HMW adiponectin through a fitness related mechanism may be more clinically relevant than reducing total adiponectin, as there is no clear relationship between low and medium molecular weight on fuel utilization or insulin sensitivity [43, 44]. Moreover, the increase in the ratio of HMW adiponectin to leptin, an indicator of adiposopathy or “sick fat”, was associated with increased fasting fat oxidation (Fig 4B) and decreased post-prandial RER (Fig 4C). While it remains possible that at least part of the changes in substrate oxidation were modified by leptin in relation to lowered dietary carbohydrate, this decrease in post-prandial RER is likely meaningful since it correlated with increased insulin sensitivity (Fig 4D). These findings are consistent with previous work from our group showing a LCD alone, or in combination with exercise increases non-oxidative glucose metabolism (i.e. glycogen storage) [19]. Hence, our findings suggest reducing adipose derived hormones and improved fuel partitioning may be a mechanism for better surgical outcomes following EX+SC.

This study has limitations that should be acknowledged. Due to the small sample size (n = 14) these findings should be considered pilot data and larger studies are needed to confirm these results. Further, inter-subject variation with this small sample size could have caused an inability to detect statistical significance, as the study was powered to test the effect of exercise on insulin sensitivity. Diet was not strictly controlled in this study. Instead, standard of care that included dietary advice was examined to gain “real-world” versus “laboratory” insight. Nearly all participants were females (n = 13). Although there are no data to suggest that females and males differ in changes in adipokines or insulin sensitivity with surgery [45], our findings may not be generalizable to males. Nevertheless, at the University of Virginia Health Systems, ~75% of patients receiving bariatric surgery are female [46, 47], and this percentage closely mirrors the national average [48]. There is potential for variation in surgical outcomes based on the type of bariatric surgery (RYGB or SG), as the average operating time and length of hospital stay is longer for patients receiving RYGB than SG, respectively [49]. Nevertheless, participants were match-paired based off surgery type to ensure the same number of participants in each treatment were receiving RYGB or SG. Although the euglycemic hyperinsulinemic clamp is considered the gold standard for determining insulin sensitivity, it is limited in providing “real-world” insight to glucose regulation. Measuring RNA with real-time PCR does not always reflect cytokine protein levels in adipose tissue, but it does allow detection and quantification of omental adiponectin and leptin expression from our cohort.

In conclusion, this pilot trial showed that adding pre-operative aerobic exercise to standard medical care decreased the length of hospital stay in patients receiving bariatric surgery compared to standard medical care alone. The reduction in length of hospital stay with EX+SC may be mediated by fitness-related adaptations including the reduction in adipose tissue derived hormones, preservation of lean mass, and enhanced metabolic flexibility. This study highlights the potential benefit of adding aerobic exercise to SC for improving metabolic health in the bariatric patient.

## Supporting information

S1 File CONSORT checklist. (DOC)

S2 File Data set. (XLSX)

S3 File Trial protocol. (DOCX)
