# Supplementary material for: Pre-operative aerobic exercise on metabolic health and surgical outcomes in patients receiving bariatric surgery: A pilot trial
Source: PLoS One. 2020 Oct 2;15(10):e0239130. doi: 10.1371/journal.pone.0239130 (PMC7531806; doi:10.1371/journal.pone.0239130)
Supplement: S3 File — (DOCX) [file pone.0239130.s003.docx]

**IRB-HSR PROTOCOL**

# Investigator Agreement

BY SIGNING THIS DOCUMENT, THE INVESTIGATOR CONFIRMS:

1. I am not currently debarred by the US FDA from involvement in clinical research studies.
2. I am not involved in any regulatory or misconduct litigation or investigation by the FDA.
3. That if this study involves any funding or resources from an outside source, or if you will be sharing data outside of UVA prior to publication that you will contact the Dean’s office regarding the need for a contract and letter of indemnification. If it is determined that either a contract or letter of indemnification is needed, subjects cannot be enrolled until these documents are complete.
4. The proposed research project will be conducted by me or under my close supervision. It will be conducted in accordance with the protocol submitted to and approved by the IRB including any modifications, amendments or addendums submitted and approved by the IRB throughout the life of the protocol.
5. That no personnel will be allowed to work on this protocol until they have completed the IRB-HSR On-line training and the IRB-HSR has been notified.
6. That all personnel working on this protocol will follow all IRB-HSR Policies and Procedures as stated on the IRB-HSR Website <http://www.virginia.edu/vprgs/irb/> and on the School of Medicine Clinical Trials Office Website: <http://knowledgelink.healthsystem.virginia.edu/intranet/hes/cto/sops/sop_index.cfm>
7. I will ensure that all those delegated tasks relating to this study, whether explicitly or implicitly, are capable through expertise, training , experience or credentialing to undertake those tasks.
8. I confirm that the implications of the study have been discussed with all Departments that might be affected by it and have obtained their agreement for the study to take place.
9. That no subjects will be recruited or entered under the protocol until the Investigator has received the signed IRB-HSR Approval form stating the protocol is open to enrollment
10. That any materials used to recruit subjects will be approved by the IRB-HSR prior to use.
11. That all subjects will sign a copy of the most current consent form that has a non-expired IRB-HSR approval stamp.
12. That any modifications of the protocol or consent form will not be initiated without prior written approval from the IRB-HSR, except when necessary to eliminate immediate hazards to the subjects.
13. Any significant findings that become known in the course of the research that might affect the willingness of subjects to enroll or to continue to take part, will be promptly reported to the IRB.
14. I will report immediately to the IRB any unanticipated problems involving risk to subjects or to others including adverse reactions to biologics, drugs or medical devices.
15. That any serious deviation from the protocol will be reported promptly to the Board in writing.
16. That any data breach will be reported to the IRB, the UVa Corporate Compliance and Privacy Office , UVa Police as applicable.
17. That the continuation status report for this protocol will be completed and returned within the time limit stated on the form.
18. That the IRB-HSR office will be notified within 30 days of a change in the Principal Investigator or of the closure of this study.
19. That a new PI will be assigned if the current PI will not be at UVA for an extended period of time. If the current PI is leaving UVa permanently, a new PI will be assigned PRIOR to the departure of the current PI.
20. All study team members will have access to the current protocol and other applicable documents such as the IRB-HSR Application, consent forms and Investigator Brochures.
21. Signed consent forms and other research records will be retained in a confidential manner. Records will be kept at least 6 years after completion of the study.
22. No data/specimens may be taken from UVa without a signed Material Transfer Agreement between OSP/SOM Grants and Contracts Office and the new institution. Original study files are considered institutional records and may not be transferred to another institution. I will notify my department administration regarding where the originals will be kept at UVa. The material transfer agreement will delineate what copies of data, health information and/or specimens may be taken outside of UVa. It will also approve which HIPAA identifiers may be taken outside of UVa with the health information or specimens.
23. If any member of study team leaves UVa, they are STRONGLY ENCOURAGED to use Exit Checklist found on IRB-HSR website at <http://www.virginia.edu/provost/facultyexit.pdf>.

The IRB reserves the right to terminate this study at any time if, in its opinion, (1) the risks of further experimentation are prohibitive, or (2) the above agreement is breached.

**Investigators Experience**

**Steven K. Malin, PhD (Contact Principal Investigator)** is Director of the Applied Metabolism & Physiology Laboratory (AMP Lab). He has published several studies that examined the effects of exercise on clinical outcomes.

**Coleen McNamara**, **MD** (**Co- Principal Investigator)** is a cardiologist and Professor in the Department of Medicine with extensive basic science experience in the study of human immune cells, specifically atherosclerosis B Lymphocyte-mediated atheroprotection. She has published studies examining B lymphocyte regulation of obesity and the metabolic syndrome and MCP-1 and diet-induced obesity.

**Peter Hallowell, MD (Co-Investigator) is the Director of Bariatric Surgery.** He has published extensively on examining the effects of bariatric surgery on clinical outcomes.

**Jenifer Kirby, MD, PhD (Co-Investigator)** is an Assistant Professor in the School of Medicine that has conducted research on lifestyle behavior modification in obese individuals.

**Arthur Weltman, PhD (Co-Investigator)** is the Director of the Exercise Physiology Laboratory. He has published numerous studies examining exercise effects on clinical outcomes.

**Eugene Barrett, MD, PhD (Co-Investigator)** is the Director of the Diabetes Research Center. He as extensive experience in the area of blood flow and insulin action.

**Zhenqi Liu, MD (Co-Investigator)** is the Chairperson of the School of Medicine Division of Endocrinology & Metabolism. He has published numerous studies related to glycemic control.

**Nicole Gilbertson, MS (Study Coordinator I)** is a PhD student in Exercise Physiology. This project will serve as work towards her dissertation.

**Julian Gaitan (Study Coordinator II)** is a Master’s student in Exercise Physiology. This project will serve as credit towards completion of his Master’s project.

Signatures

**Principal Investigator**

____________________________ ____________________________ _______

Principal Investigator Principal Investigator Date

Signature Name Printed

The Principal Investigator signature is ONLY required if this is a new protocol, a 5 year update or a modification changing the Principal Investigator.

**Department Chair**

BY SIGNING THIS DOCUMENT THE DEPARTMENT CHAIR AGREES:

1. To work with the investigator and with the board as needed, to maintain compliance with this agreement.
2. That the Principal Investigator is qualified to perform this study.
3. That the protocol is scientifically relevant and sound.

___________________________ _______________________ _________

Department Chair or Designee Department Chair or Designee Date

Signature Name Printed

# Brief Summary/Abstract

Obesity is a major health concern that has been associated with an estimated 2.8 million deaths worldwide each year. The number of individuals considered obese with a Body Mass Index (BMI) above 30 kg/m^2^ has grown to more than 500 million. The increased morbidity and mortality associated with obesity stems from a long list of comorbidities, including hypertension, coronary artery disease, stroke, cancer, and type 2 diabetes (T2D). Bariatric surgery is an emerging intervention that has been used frequently to induce weight loss for obese individuals and it has been shown to improve glycemic control and insulin resistance in people at risk for type 2 diabetes. Beyond weight loss and improved glycemic control, surgery may lead to healthy improvements in inflammation, immune cells and vascular health. It is already known that exercise and weight loss from lifestyle modification can improve glycemic control, insulin resistance, levels of inflammatory cytokines, gut bacteria and arterial stiffness. However, no work has been done to examine a combination of bariatric surgery and pre-surgery lifestyle modification with diet and exercise. Recent work by our team has evidence demonstrating that health status pre-surgery has an impact on post-surgery outcomes. Such findings suggest that improvements in health status from exercise and/or caloric restriction before surgery may improve surgery outcomes as well as surgery-induced health outcomes. To date, no study has systematically examined the role of diet and/or exercise on the prevalence of surgery complications or on post-surgery weight loss, glycemic control, and insulin resistance. Moreover, no work currently exists with lifestyle modification, with or without bariatric surgery on adipose tissue derived inflammation related to immune cells or gut bacteria. Therefore, the purpose of this study is to investigate the effect of pre-surgery lifestyle intervention with diet and/or exercise on bariatric surgery outcomes. To test this objective, subjects will participate in a match paired study, based on BMI. Subjects will undergo testing of blood chemistry and related measures of health before and after lifestyle intervention, as well as 4 weeks post-bariatric surgery. The subjects will be match paired to standard care or exercise until these interventions are filled. Once standard care and exercise interventions are full subjects will then be match paired to exercise plus diet and then diet last. After the intervention all subjects will receive surgery. Post surgery outcomes will be assessed by examining surgery operating time, changes in blood chemistry, adipose tissue biopsies and other measures indicative of glucose and vascular health.

# Background

1. **Provide the scientific background, rationale and relevance of this project.**

Obesity has reached epidemic proportions in the United States (US), as ~34% of the adult population in the US is obese, defined as having a body mass index > 30 kg/m^2^. This is problematic because excess body fat has a strong association with increased mortality and is considered a chief risk factor promoting the development of chronic diseases such as atherosclerosis and type 2 diabetes (T2D). Obesity establishes a link between chronic diseases when in part elevations in circulating free fatty acids (FFA) and adipose tissue derived cytokines induce insulin resistance via inflammatory pathways (13, 14). Insulin resistance in turn promotes the accumulation of visceral abdominal fat and exacerbates hyperglycemia, unbalanced adipokine profiles (adiponectin, MCP-1 and leptin), increased white blood cells, elevated LDL cholesterol, beta-cell dysfunction, and arterial stiffness (5-7, 13-17). Additionally, dysbiosis of the gut microbiota is linked to obesity and atherosclerosis (38-40). Thus, interventions targeting reductions in excess fat are likely to lower overall cardiovascular disease (CVD) risk.

Bariatric surgery is currently used as a metabolic tool to treat obesity in large part because the Center for Diseases and Control predicts that obesity will reach 42% by 2030 in the US, and many metabolic abnormalities are improved. Roux-en-Y gastric bypass (RYGB) surgery remains the most commonly performed bariatric procedure, although the use of sleeve gastrectomy (SG) as risen in the last few year because of its efficacy for weight loss and reduced surgical complications compared with RYGB. Subsequently, RYGB and SG make up ~85% of all bariatric surgeries. Weight loss is the primary motive for bariatric surgery in obese patients. The Swedish Obesity Study, a large prospective intervention-based trial, found that bariatric surgery interventions induced 23%, 17%, and 18% body weight reduction at 2, 10, and 20 year follow-ups respectively (18). Post-surgically, RYGB has been linked to changes in gut microbial diversity that correlate with reduced adiposity, hyperglycemia, inflammatory cytokines, and inflammatory gene expression in white adipose tissue (41, 42). Bariatric surgery is also recognized as a treatment—some call it a “cure”—for T2D. In fact, the ability of bariatric surgery to reverse T2D has been recognized for more than 20 years. That notion began with a classic trial by Pories et al. who reported that 139 of 141 patients with T2D achieve normalized glucose tolerances within 10 days following RYGB (19. Since then, a meta-analysis of RYGB research has shown a mean rate of T2D remission of 83.7% (95% CI 77.3—90.1%) (20). Despite the overall success of these procedures, the durability of long-term weight loss and CVD risk reduction is highly variable as up to 30-70% of people regain weight and metabolic health issues within 2-3 years post-surgery (7) Addressing these health in the bariatric patient requires a novel approach targeted at optimizing metabolic health.

There is a growing body of evidence suggesting that pre-surgical health status—even among obese diabetic patients—predicts the likelihood of success from surgery. Recent work on this topic has attempted to predict T2D remission or non-remission following RYGB surgery by looking at patients’ pre-surgery health status and their subsequent surgery outcomes (2, 21-23). Preoperative duration of T2D greater than 10 years and poor preoperative glycemic control with an HbA1C > 10 have been cited as strong predictors of T2D non-remission (21, 22). Preoperative waist circumference—which has a strong association with accumulation of lipotoxic visceral abdominal fat—has also been linked with T2D remission status post-RYGB (23, 24). Additionally, patients who use oral hypoglycemic medications, rather than insulin injection, are more likely to achieve T2D remission (23). Recent investigations from our team have examined the metabolic determinants of T2D non-remission status after bariatric surgery (2). The chief characteristic of T2D severity is impaired pancreatic 𝛽-cell insulin secretion for the prevailing degree of insulin resistance (25, 26). The mechanism for this impairment is not completely understood, but a contributing factor in terms of obesity is that excess body fat leads to dysregulation of adipokines (leptin, PAI-1, TNF-α, adiponectin), which impair insulin signaling and secretion pathways (14, 15, 27). As such, it follows that differences in adipokine and inflammatory marker profiles, which improve concurrently with weight loss (28), may predict T2D remission or non-remission status. Malin et al. found that greater increases in circulating adiponectin and decreases in C-reactive protein (CRP) from baseline (pre-surgery) characterize T2D remission status 2 years after surgery (RYGB or SG; 2), independent of pre-existing BMI, age, sex and hyperglycemia (4, 5, 11). **Taken together, immune related inflammation appears to be a key determinant of bariatric surgery induced CV health.**

Vascular arterial stiffness is powerful independent predictor of CVD events (29), and several lines of research support the notion that chronic hyperglycemia and/or postprandial hyperglycemia per se contributes to the pathogenesis of atherosclerosis via impairments in vascular endothelial cell function (30-31). In fact, each component of the vasculature impacts oxygen, hormones and substrate delivery to metabolically active tissues. Vascular stiffness is driven by increased content and cross-linking of collagen, loss of elastin and by increased smooth muscle tone in the conduit artery wall. Changes in arterial expression, architecture and bioactivity of structural proteins may play a key role if stiffening of the artery. Nitric oxide (NO) and insulin are a potent vasodilator with atheroprotective actions in conduit vessels and important effects on resistance and terminal arterioles that regulate tissue perfusion. Consistent with alterations in adipose tissue derived inflammatory markers, immune cell activity has been associated with arterial stiffness and the progression of atherosclerosis (33, 34). Weight loss leads to improvements (reduction) in the arterial augmentation index (AIx), measured by applanation tonometry at the radial artery. Furthermore, a reduction in AIx has been associated with improvements in immune cell function and adipose tissue gene expression. For example, a 24 week study from Samaras et al. (33) that included a 12 week caloric restriction diet and sleeve gastrectomy (SG) found significant correlations between a reduction in AIx and a fall in the number of T-lymphocytes and CD3 cells. Reductions in AIx were also associated with reductions in cell surface expression of monocyte adhesion molecules, granulocyte activation molecules, and T-lymphocyte IL-2 receptors. However, this existing literature is limited by a lack of control groups to differentiate the independent effects of lifestyle change from the effects of bariatric surgery. Furthermore, existing literature has not evaluated if improvements in vascular health are seen in vitro before whole body adaptations occur. Interestingly, pre-weight loss intervention health markers such as baseline insulin resistance and CD3 lymphocyte numbers may predict greater improvements in AIx


(33). Another study examining cardiac and vascular morphological changes following SG found that aortic elasticity and LV diastolic function were improved compared to baseline at 6 and 12 month follow ups (35).

While bariatric surgery is an effective therapeutic option for inducing marked weight loss and remission of obesity-related diseases (e.g. cardiovascular disease (CVD)) (7), ~30-50% of patients who undergo this very invasive procedure redevelop many of their CVD complications (i.e. insulin resistance, dyslipidemia, hypertension, etc.) (5). Obviously, identifying pre-operative biomarkers associated with recurrence of cardio-metabolic abnormalities that could be used to counsel patients and develop approaches aimed at further enhancing successful weight loss for those undergoing bariatric surgery has tremendous clinical value. To date, nearly all clinical interventions aimed at sustained weight-loss and lower disease risk in response to bariatric surgery are applied AFTER surgery (3, 12) *In contrast,* there is no standardized medical therapy BEFORE bariatric surgery that is designed to minimize surgical-induced complications and/or enhance CVD risk reduction. Patients are typically recommended to lose 5-10% of their initial presenting weight prior to bariatric surgery based on the hypothesis that patients would have less surgical complications. Indeed, patients who are instructed to lose ~5-10% of their initial weight prior to surgery by CR had shorter operating room time, less intraoperative blood loss, less postoperative pain, and improved short-term postoperative weight loss (10). While CR (~800-12000 kcal/d) for ~4 weeks accomplished this goal in obese patients, the mechanism explaining reduced metabolic risk of surgery is unknown. In addition, no work has studied pre-operative Ex on reducing CVD risk. This is clinically important since CV mortality is significantly lowered by Ex through gains in aerobic fitness (i.e. VO2max) (2) and exercise improves quality of life (1) In fact, several studies by Malin et al. show that Ex drives reductions in insulin resistance and CVD risk through in part lowering adipose-derived inflammation (6, 8, 9). As a result, lifestyle intervention is a reasonable strategy to target cardiometabolic risk in obese patients who may undergo bariatric surgery (36, 37). **To date, however, no study has determined the role of immunometabolism before or after exercise (Ex) and/or caloric restriction (CR), with and without bariatric surgery. Secondarily, no work has investigated if exercise-induced health change before surgery can 1) reduce disease risk pre-operatively and 2) improve responsiveness to bariatric surgery. Together, investigation on how exercise and/or diet impacts immunometabolism and cardiometabolic risk in obese patients will lead to a better understanding of how to develop metabolic fitness programs for people undergoing bariatric surgery.**

# Hypothesis to be Tested

The central hypothesis is that pre-operative metabolic health is an important predictor of bariatric surgery effectiveness. We hypothesize that there is an adipose-derived immunosignature that predicts post-operative recurrence of obesity related CVD abnormalities and that targeting reductions in obesity BEFORE surgery will enhance the success of bariatric surgery on this disease risk. We expect that the combination of exercise and diet will be more successful at improving insulin sensitivity and reducing immune cell dysfunction compared to diet alone as well as expect that exercise will be superior to standard of care alone. These separate studies are expected to provide unique insight to the mechanism by which diet and/or exercise impact immune metabolism in obese patients, and this will shape the development of future studies in bariatric patients per se.

Primary Objectives:

1. **Ascertain the effects of Ex+CR vs. CR in obese subjects and Ex vs. standard of care (SC, i.e. control) BEFORE surgery on cardiometabolic risk.**
2. **Examine the effects of Ex+CR vs. CR in obese subjects and Ex vs. standard of care (SC, i.e. control) BEFORE surgery on surgical complications and quality of life.**
3. **Determine if circulating immunophenotyping after Ex+CR vs. Ex vs. CR vs. SC correlate with immune composition of adipose cell depots (omental and subcutaneous) at the time of surgery or post-intervention testing**.

**Study Design: Biomedical**

**1. Will controls be used?**

Yes

**►IF YES, explain the kind of controls to be used.**

Subjects will have pre-intervention testing to determine efficacy of exercise and/or diet on insulin sensitivity and immune health.

1. **What is the study design?**

Matched pair, prospective design. Bariatric patients will be match paired to the exercise or standard medical care group. Subjects not undergoing bariatric surgery will be randomized to calorie restriction or calorie restriction + exercise groups.

1. **Does the study involve a placebo?**

No

# Human Participants

**Ages:** 18-70 years

##### Sex: Men and Women

**Race:** All

**1. Provide target # of subjects (at all sites) needed to complete protocol.**

A total of **60 subjects** will be needed to complete the study in order to determine statistically significant results.

**2. Describe expected rate of screen failure/ dropouts/withdrawals from all sites.**

Based on prior work by our group, we anticipate that 20% of subjects who receive randomization (i.e. treatment) in the study will withdraw/drop out early.

For individuals not receiving bariatric surgery, we anticipate that 30% of subjects who are initially recruited to participate in the screening visits will not meet the criteria for inclusion in the study (i.e. not passing physical examination, abnormal ECG, abnormal exercise test, abnormal blood chemistries, outside of BMI range, pregnancy). Therefore, we expect to screen 38 individuals not receiving bariatric surgery for participation in the study. Of the 38 subjects screened, 28 will undergo the treatment. Accounting for dropouts, 24 subjects will complete the study.

For individuals receiving bariatric surgery, we anticipate 20% of subjects who are initially recruited to participate in the screening visits will not meet the criteria for inclusion in the study (i.e. not passing physical examination, abnormal ECG, abnormal exercise test, abnormal blood chemistries, outside of BMI range, orthostatic intolerance). Therefore, we expect to screen 20 individuals receiving bariatric surgery for participation in the study. Of the 20 subjects screened, 16 will undergo the treatment. Accounting for dropouts, 12 subjects will complete the study.

**Thus, we expect to screen 60 subjects of whom 50 will receive treatment and 40 will complete the study.**

**3. How many subjects will be enrolled at all sites?**

We expect to enroll 60 subjects of whom 50 will receive treatment and 40 will complete the study.

##### 4. How many subjects will sign a consent form under this UVa protocol?

A total of 60 subjects will sign the consent form.

1. **Provide an estimated time line for the study.**

We anticipate this study will take approximately 18-36 months to meet 100% enrollment. Each participant will be enrolled for a total of approximately 12 weeks from the time of screening to study completion. Our team will interact with 4-5 subjects at one time. As a result, approximately 8-12 subjects per year will be enrolled.

# Inclusion/Exclusion Criteria

**1. List the criteria for inclusion**

- Males and Females, 18-70 years of age
- BMI >30 and <70 kg/m^2^
- Sedentary (Not currently participating in exercise training: >30 min. of physical activity per day, >3 days/week)
- HCT for women > 36%, Men >38%
- Non-pregnant (women).-self reported
- Smoker (if bariatric surgery patient) or non-smoker (enrolled for the dietary portion of the study)
- Has the ability/willingness to participate in the study and agree to any of the arms involved in the study.
- No prior surgical procedure for obesity with the exception of a laparoscopic adjustable gastric banding (LAGB) under the condition that the band had not been adjusted in ≥ 1 year

**2. List the criteria for exclusion**

- Currently participating in exercise training: >30 min. of physical activity per day, >2 days/week)
- Cigarette smoking (presently or in the past 6 months), drug or alcohol abuse
- Pregnancy or breastfeeding
- History of congestive heart failure, ischemic heart disease, severe pulmonary disease.
- History of cancer (within 5 years)
- Diagnosed as insulin-dependent diabetes.
- Change in psychotropic medication dosage in past six weeks
- AST or ALT > 3 times normal range
- Currently involved in any active weight loss treatment program (other than self-directed attempt at calorie restricting diet) or lean patients (BMI <29 kg/m^2^).
- Current purging behavior occurring > once a week over the past six weeks (self-induced vomiting for weight control purposes, laxative or diuretic abuse)
- Revisional bariatric procedures including a RYGB reversal to a SG or a LAGB revision to a SG or RYGB if the band has been adjusted in ≤ 1 year.
- Active psychotic illness, including bipolar affective disorders.
- Evidence of current suicidality or homicidality
- Conditions associated with significant cognitive dysfunction (e.g. dementia) or medical instability that puts the participant at risk
- Contraindication to exercise (severe/uncontrolled CVD; inability to walk 2 blocks, bone or joint problems )
- Allergy to “caine” family drugs (e.g. lidocaine).
- Currently taking active weight suppression medication (e.g. phentermine,bupropion SR, topiramate).
- On medication known to cause substantial weight gain (e.g. atypical antipsychotics such as olanzapine, sodium valproate, steroid therapy). This would not include medications commonly used in this population that usually result in only mild weight loss (e.g. SSRIs).

**3. List any restrictions on use of other drugs or treatments**.

- Medication or food supplement known/thought to affect insulin sensitivity or endothelial function (TZDs, sufonylureas, biguanides, alpha-glucosidase inhibitors, phosphodiesterase inhibitors, beta-blockers, alpha-blockers, ACE-inhibitors, ARB’s, fibrates, glucocorticoids, fish oil, allopurinol, Vitamin E, Vitamin C).
- Subjects cannot ingest medications or food supplements (e.g. statins, dietary supplements, etc.) 24 hours before testing.
- Subjects cannot drink alcohol 24 hours before testing.
- Subjects cannot eat or drink foods that contain caffeine 24 hours before testing.
- Subjects must refrain from any structured exercise for 24 hours prior to testing and limit exercise to their normal everyday activities.
- Currently taking active weight suppression medication (e.g. phentermine,bupropion SR, topiramate)
- On medication known to cause substantial weight gain (e.g. atypical antipsychotics such as olanzapine, sodium valproate, steroid therapy). This would not include medications commonly used in this population that usually result in only mild weight loss (e.g. SSRIs).

# Statistical Considerations

1. **Is stratification/randomization involved?**

No. Subjects will be match paired.

**►IF YES, describe the stratification/ randomization scheme.**

**►IF YES, who will generate the randomization scheme?**

_____

**2. What are the statistical considerations for the protocol?**

This study is a between-within subjects repeated measures design in which insulin sensitivity will be measured before and after interventions across 4 experimental conditions. The outcome measures to be assessed are as follows:

**Outcome assessments pertaining to the primary objective:**

- 1. Assess the absolute mean difference in the 3-hr postprandial glucose AUC between the 2 different conditions undergoing bariatric surgery and the 2 different groups not undergoing bariatric surgery.
  2. Assess the absolute mean difference in the 3-hr postprandial insulin AUC between the 2 different groups undergoing bariatric surgery and the 2 different groups not undergoing bariatric surgery.
  3. Compare the postprandial insulin sensitivity across conditions (as measured by the insulin sensitivity index- ISI; a calculated value based on the glucose/insulin values obtained from the MMTT or OGTT)
  4. Compare the mean change in body fat between groups.
  5. Compare the mean change in body weight between groups.
  6. Assess the change in cardiorespiratory fitness between groups.
  7. Determine and compare the immunophenotype from blood between groups.
  8. Relate the insulin sensitivity responses to the immunophenotyping from blood via correlations.
  9. Relate the pulse wave velocity responses to the insulin sensitivity and immunophenotyping under each condition by calculating a 3-hour pulse wave velocity AUC.
  10. Relate the augmentation index responses to the insulin sensitivity and immunophenotyping under each condition by calculating a 3-hour pulse wave velocity AUC.

**Outcome assessments pertaining to the secondary objectives:**

1. Compare differences in omental and subcutaneous immune cell composition levels before and after surgical lifestyle interventions.
2. Compare omental and subcutaneous immune cell composition levels to circulating levels after the intervention at the time of bariatric surgery.
3. Compare differences in mean plasma adiponectin, MCP-1, leptin and C-RP levels before and after the interventions with insulin sensitivity, and immune cells.
4. Compare differences in gut bacteria before and after the interventions and associate with changes in insulin sensitivity and immune cells.
5. Identify if changes in pre-operative insulin sensitivity, weight loss, inflammation (e.g. adiponectin, etc.) and immune cells predict changes in bariatric responses 1 month post-op.
6. Correlate at the time of bariatric surgery insulin sensitivity, immune cells, and weight loss with operating time.

**3. Provide a justification for the sample size used in this protocol.**

*No prior work has studied adipose immune cell health in bariatric patients before or after surgery or in response to Ex+CR.* As a result, data collected in the proposed study will serve as preliminary work to determine power analysis for future studies and NIH proposed grants.

Nonetheless, the appropriate sample size required for significant differences following Ex+CR testing and surgery was, however, determined from our preliminary work (6, 11) on insulin resistance given it’s direct relation to adiposity-related immunity. A delta of 2 (Pre = 3.5 vs. Post = 5.5 surgery), SD of 1.2 with 80% power and an alpha of 0.05 indicates that **5** subjects are needed to show insulin resistance differences post-surgery, which is consistent with Ex+CR reducing insulin resistance in **6** people (Pre = 2.5 vs. Post = 4.9), SD of 1.5 at 80% power. **Thus, assuming a 20% dropout rate, 8 are required per group** **undergoing bariatric surgery.**

In contrast, subjects undergoing diet+exercise vs. diet intervention will require more subjects. This is based on an diet+exercise published study (43) on insulin resistance and energy deficit (i.e. weight loss). A delta of 3.1kg (Exercise = 3.7kg vs. Exercise+diet = 6.8kg), SD of 3.1kg with 80% power and an alpha of 0.05 indicates that **10** subjects are needed to show energy deficit differences. **Thus, assuming a 20% dropout rate, 14 will be required per group** **NOT undergoing bariatric surgery.**

**4. What is your plan for primary variable analysis?**

***Analysis between weight loss, insulin sensitivity and immune health:***

Data will be analyzed using R (Vienna, Austria, 2011). A two-way repeated measures analysis of variance (ANOVA) will be used to assess time course differences (group x test) for **AIM 1** and **AIM 2**. A repeated measures ANOVA will also be used to assess differences in adipose cell depot among groups (**AIM 3**). Bonferonni post-hoc analysis will be used to determine group differences and paired t-tests will be used to assess within treatment effects. Linear regression analyses will be used to identify relationships between respective variables. A *P*<0.05 will be considered statistically significant. Data will be expressed as mean ± SEM.

**5. What is your plan for secondary variable analysis?**

The same statistical approach to primary variable analysis will be conducted.

Bivariate regression analysis to understand the relationship between blood and urine MPs to inform future studies which tissue to collect from. Currently, it is not understood how MPs collected in the blood relate to MPs collected in the urine.

**6. Have you been working with a statistician in designing this protocol? NO**

**7. Will data from multiple sites be combined during analysis? N/A**

# Biomedical Research

**1. What will be done in this protocol?**

This study will determine cardiorespiratory fitness (VO2max test, 6-minute walk test), body composition analysis, blood chemistry during a mixed-meal tolerance test, vascular function (pulse wave velocity and augmentation index), immune cell phenotyping, and adipose tissue gene expression of inflammatory markers before and after exercise and/or diet compared with control both before and after bariatric surgery. Below describes each assessment.

**2. List the procedures, in bullet form, that will be done for RESEARCH PURPOSES as stipulated in this protocol.**

**** All procedures/assessments are being done for research purposes only****

Each subject will visit UVA for around 15 visits that include: the CRU 5 times – one preliminary screening visit, one visit to obtain baseline fitness and anthropometric data, and one visit per experimental condition (described below), up to 8 exercise sessions in Memorial Gymnasium with exercise physiologists, and/or about 4 visits for dietary pick up at Memorial Gymnasium or the CRU (which ever is more convenient to the subject).

**Outpatient Screening Visit #1**: **(this visit will last about 2 hours)*:***

- Potential subjects will report to the CRU at least 12-hours in the post-absorptive state (nothing but water after ~7-8 PM the night before). Participants will be asked to report to the CRU between 7 and 8 AM (i.e., 12 hours after their last meal).
- Participants will be consented and then carefully queried to ensure that they meet the pre-screening eligibility criteria as described in the inclusion/exclusion criteria using the Screening Questionnaire (that includes the Physical Activity Readiness Questionnaire or PAR-Q: This is a checklist of 9 questions ensuring subjects can participate in cardiovascular exercise. This questionnaire is routinely used by the American College of Sports Medicine to assess safety for exercise in at risk populations).
- Eligible persons will provide written informed consent prior to participating in any

experimental procedures.

- Vital signs, height and weight will be measured.
- Prior to testing, A needle will be inserted into a vein in either the forearm or back of your hand to take a blood sample. .
- Blood will be drawn for CBC, liver function tests, HbA1c, HCT for women > 36%, Men >38%, BUN, fasting blood glucose and fasting lipid profile. For females, a serum HCG will be drawn.

This visit will take approximately 2 hours and approximately 9 cc of blood will be drawn for men and 12cc for women.

**Subjects meeting the above criteria will be continue on and attend the 2^nd^ study test visit. This test will be conducted on a separate date and be within 1 month after the above screening visit):**

***Phase 2: SCREENING AND STUDY TESTING PROCEEDURES***

**Study Test Visit 2 (this visit will last about 2 hours)*:***

**Subjects must fast (not eat) for 4 hours before this visit.**

The second screening visit will start with a:

- Review of the subject’s medical history
- Physical exam and vital signs (blood pressure, heart rate and resting electrocardiogram)
- If subjects are women who are able to bear a child, they will have a urine pregnancy test that must be negative in order to participate.

The purpose of the physical exam is to determine if subjects are eligible for the exercise portion of the study and that it is safe for them to participate. The pregnancy test must be negative in order for the subjects to participate. If subjects continue to qualify for the study the remainder of the visit will involve the study procedures described below:

**A. Body Composition**:

- Subjects must be fasting for at least 4 hours before this procedure.
- Subjects weight will be measured without shoes and while wearing minimal clothing.
- Subjects waist and hip circumference ratio will be obtained with a tape measure to determine how much fat is located in the central part of the body.
- We will measure the total amount of fat and muscle in your body with the BodPod® in the Exercise Physiology Core Lab.
- We will provide a swimsuit and swim cap for you to wear during this procedure to help standardize your results.  The swimsuit and swim cap are provided to get accurate results.  You will not get wet at anytime during this measurement.
- The research team will also measure the regional amount of fat and skeletal muscle in the subjects’ bodies with the InBody®. This testing will be conducted in Memorial Gymnasium and a team member will walk the subjects over to the location.
- The InBody® procedure requires subjects to stand on a platform and hold a handrail. This test using a low-level, safe, electrical current through the body. The current travels at a different rate through the various body tissues, which then allows a calculation of body fat and muscle.
- The entire InBody® procedure takes approximately 15 minutes.
- Weight will also be obtained on a Bod Pod scale without shoes and while wearing minimal clothing in the Exercise Physiology Core Lab.
- Body composition (%body fat) will be determined by air displacement plethysmography. This requires that the participant sit in a 750-liter volume, dual chamber fiberglass shell and rest quietly for a few minutes. The Bod Pod has a window that enables the participant to see out of the chamber at all times, thus minimizing the risk of claustrophobia. Bod Pod measurements take approximately 5-7 minutes total time to complete.

**B. Treadmill or cycle ergometer exercise testing for cardiovascular fitness (i.e. VO2max)**:

- Subjects will be asked to perform a maximal treadmill or cycle ergometer exercise test in the Exercise Physiology Core Laboratory in the School of Medicine. The treadmill test will begin with low speed on the treadmill, and the speed and/or elevation will increase gradually every 2 minutes. You will complete the treadmill test if undergoing bariatric surgery. However, if you are not undergoing bariatric surgery, then you will perform the cycle ergometer test. The cycle ergometer test will begin with low resistance on the bicycle wheel, and the resistance will increase gradually every 3 minutes. Subjects will be asked to go as long as they can, that is, until they feel exhausted.
- During the test, subjects will have continuous electrocardiogram (ECG) heart monitoring and blood pressure monitoring by professionally qualified exercise physiologists of the Exercise Physiology Core Lab. All testing procedures follow the American College of Sports Medicine Guidelines.
- During exercise testing researchers will also measure the subjects’ metabolic rate, a measure of the amount of energy it takes to burn calories. During exercise subjects will wear a facemask that is connected to a special device (metabolic cart) that measures subjects’ exhaled carbon dioxide (CO_2_) and oxygen. These measurements are then used to calculate the amount of calories they burn.
- During the exercise test, the subject will wear a mask over their nose and mouth. This allows the researchers to measure how much oxygen the subject is using and will tell the researchers what the subject’s fitness level is.
- The visit ends after the subject completes the exercise test. The subject will receive the results of their test after the completion of the study.
- Subjects will also be provided with an **accelerometer** and instructions on how to use the accelerometer. The accelerometer is a small device, similar to a pedometer, which is worn on the belt and records the amount of activity performed. Data will be downloaded from it on a weekly basis.

**C. Timed Walk Test (ie. 6-Minute Walk Test):**

- Subjects will be asked to complete a timed 6-minute walk test (6-MWT) on a reserved basketball court in Memorial Gymnasium under supervision of study personnel after physician clearance. Subjects will be instructed to wear comfortable shoes and can take medications as usual.
- The basketball court will be clearly marked every 3 meters for 30 meters total.
- Monitoring equipment used include a stop watch, heart rate monitor, blood pressure cuff, and Borg scale for evaluation of perceived exertion. Prior to the walk test, resting heart rate, blood pressure, and perceived exertion will be measured to ensure the subject is within the American College of Sports Medicine’s recommendations for exercise.
- Individuals will be instructed to walk as quickly as they can up and down the basketball court for 6 minutes so that they cover as much ground as possible. Subjects will be instructed that they can use ambulatory devices (ie. cane or walker) and can stop to take rests as needed.
- Subjects will be updated on the time remaining every minute.
- Immediately at the end of the test and blood pressure, heart rate, and perceived exertion will be measured. It will be marked where the subject stopped after 6 minutes and total distance traveled will be recorded.
- Heart rate and blood pressure will be measured periodically until levels return to resting value. At this point the subject will be allowed to leave the facility.

**D. Physical Activity Questionnaire:**

- *Minnesota Leisure Time Physical Activity:* This is a checklist of 60 physical or recreational activities subjects may have participated in over the last 12 months.

**Study Testing Visits 3-6: (each visit will last about 6 hours):**

**The following information will be provided to the subject**

- While the subject is in the study, they will be asked to maintain their normal physical activity level.
- Subjects will be asked to follow a special diet for 1 day before study testing visits 3, 5 and 7. The diet will require subjects to eat at least 200 grams of carbohydrate each day while limiting the amount of fat ingested. Subjects will be given a chart to help record their carbohydrate and food intake and must return the chart on the day of their admission.
- **Subjects must not drink alcoholic or caffeinated beverages for 24 hours before the study testing visit begins** (i.e. after 7am the day before the test).
- **Subjects** **must not use allergy or pain-related medicines (over-the-counter or prescription**) for at least 5 days prior to each admission.
- **Subjects must not use antibiotics for 3-4 weeks before study testing visits.** If subjects have been prescribed an antibiotic 3-4 weeks before the study or are prescribed an antibiotic during the study they should inform the investigators. This may result in an inability to collect fecal matter for gut microbiota analysis.
- **Subjects must not use statins, glucose lowering medication, or antioxidant dietary supplements 24 hours before the study testing.**
- **Subjects must not perform any vigorous exercise for 48 hours prior to each test day**.
- The tests must be performed in the fasted state, so subjects may not eat or drink anything (except water) after about 7pm the night before.
- Subjects will be asked to report to the CRU by approximately 7:00am on the morning of each test.
- If a subject is a pre-menopausal woman, they will be asked to report to the CRU to provide a urine sample for a pregnancy test. The test must be negative in order for them to continue their participation.

**Urine Collection**:

- Subjects will be provided with a plastic container and asked to collect their first morning urine void. The amount and time urine collection occurs will be recorded and analyzed for nitrogen. In non-bariatric patients, subjects urine collection will be recorded, divided, and analyzed for nitrogen and urine microparticles.

**Fecal Collection:**

- Subjects will be provided with a sterile commode container and asked to collect fecal matter. The amount of fecal matter collected will be recorded and analyzed for gut microbiota.

**Blood draws**:

- In the morning of the 4 study testing days, an IV catheter will be inserted into a vein in either the forearm or the back of the subject’s non-writing hand.
- The IV catheter will be in their arm for the remainder of each visit (about 5 hours), and removed before they leave the CRU.
- During the 4 study visits their blood will be drawn as follows:
  - Fasted
  - Every 30 minutes up to 180 minutes after their drink the beverage
- On each visit, a total of approximately 5-6 tablespoons of blood will be drawn. The blood we take will be tested to measure glucose, insulin, and substances that will help estimate blood glucose control and vessel health.
- A sweet mixed-meal that contains 4 oz of boost meal shake will start the mixed-meal tolerance test (MMTT) for bariatric patients. An oral glucose tolerance test (OGTT) will be given to subjects not undergoing bariatric surgery.
- Non-bariatric patients will have blood drawn (6 ml per draw; 2 draws) to assess blood microparticles, a new biomarker to assess cardiovascular disease risk, at visit 3 and visit 6 (or 18).
- Once the subject drinks the glucose beverage they will return to bed for the remaining blood draws (3-hours)
- When the blood draws are completed they will be fed lunch and discharged to go home.

**Glucose Metabolism tests (i.e. blood sugar):**

*Mixed-Meal Tolerance Test (MMTT) – for subjects undergoing bariatric surgery*

- The MMTT is used to help determine how quickly sugar is removed from the blood and insulin levels rise. After the blood sample is taken for the fasting blood sugar test, the subject will drink a meal shake. Five more blood draws will be taken at 30, 60, 90, 120, and 180 minutes after they receive the drink. The total amount of blood taken is approximately four teaspoons. During this MMTT, the subjects’ metabolic rate, a measure of the amount of energy it takes to burn calories, will be performed at minutes 0, 60, 120 and 180. During this metabolism measure, subjects will wear a canopy that is connected to a special device (metabolic cart) that measures subjects’ exhaled carbon dioxide (CO_2_) and oxygen. These measurements are then used to calculate the amount of calories they burn and will be performed by trained exercise physiologists.

*Oral Glucose Tolerance Test (OGTT) – for subjects not undergoing bariatric surgery*

- The OGTT is used to help determine how quickly sugar is removed from the blood. After the blood sample is taken for the fasting blood sugar test, they will drink a sugary solution.  Five more blood draws will be taken at 30, 60, 90, 120, and 180 minutes after they receive the drink. The total amount of blood taken is approximately 1 1/3 tablespoons. During the OGTT test, the subjects’ metabolic rate, a measure of the amount of energy it takes to burn calories, will be performed at minutes 0, 60, 120 and 180. During this metabolism measure, subjects will wear a canopy that is connected to a special device (metabolic cart) that measures subjects’ exhaled carbon dioxide (CO_2_) and oxygen. These measurements are then used to calculate the amount of calories they burn and will be performed by trained exercise physiologists. The subject’s appetite response to the OGTT will be tested before and after this test.

**Vascular tests:**

*Blood pressure:*

- - The subject’s blood pressure will be taken: before the MMTT and every hour afterwards (at about the same time their blood is being drawn). This test takes about 5 minutes.
- **Augmentation Index (AI)** This measures the pulse wave of the vessel and its characteristics, which is an assessment of the stiffness of the aorta. Researchers will use an aplanation tonometer from SphygmoCor. This is done by placing a blood pressure cuff around the subject’s arm and measuring several waveforms. This will take 2-5 minutes and is comparable to routine blood pressure readings.
- **Pulse Wave Velocity (PWV)** This measures the time difference between the pulse wave at the carotid artery and the pulse wave at the femoral artery. This will allow researchers to assess the “stiffness” of the larger vessels. The shorter the time difference, the stiffer or less elastic the vessels are. Researchers will use the same device as described above. Specifically, a probe that looks like a “fat” pen will be placed over the carotid artery at the neck and a blood pressure cuff will be placed around the upper leg. Researchers measure the time between the heartbeat and when the probe picks up the pulse wave with the tonometer. This will take about 10-15 minutes.
- **Brachial Artery Flow Mediated Dilation (FMD)** Brachial artery diameter is measured using ultrasound at baseline and then after five minutes of forearm ischemia. The subject will supine in bed.  The first measurement is taken (velocity and diameter) and then a blood pressure cuff placed around the forearm is pumped to 200 mm/Hg and held for 5 minutes.  The brachial artery will be observed with ultrasound as the BP cuff is deflated and for 2 minutes post cuff deflation.  This will take approximately 10 minutes. We will perform this procedure just before the beverage, and then again at 1, 2, and 3 hours after the drink.

**Adipose Biopsy***:*

- Adipose biopsy samples will be taken during MMTT/OGTT (Optional). The biopsy involves removal of a very small piece of fat tissue beneath the subject’s skin (referred to as subcutaneous fat) and is about the amount of a pencil eraser. This is performed by way of a needle inserted into your belly area near your navel (belly-button) through a quarter inch skin incision. Local anesthesia will be used to numb the area where the incision will be made. Fat biopsies will be performed on alternate sides of the stomach.
- The abdominal area will be cleansed with an iodine solution. Once thoroughly cleansed and dry, a small amount of numbing (about less than ½ teaspoon of lidocaine), will be injected into the area to be biopsied.
- Once the area is sufficiently numbed, a small incision will be made (approximately 5mm, or less than ¼ of an inch) and a biopsy needle will be inserted in order to obtain approximately 10 grams. Once the biopsy has been completed, slight pressure will be applied to the biopsy area to minimize any bleeding. The area will then be cleansed and a special bandage will be applied to the biopsy site. Subject’s will be asked to keep this bandage on the area for the next 24 hours to reduce risk of bleeding.

**Questionnaires:**

- *Appetite Questionnaire:* This questionnaire examines the subject’s response to the food they eat. They will fill this out before the MMTT or OGTT and 2-hours after consuming the beverage.
- *Daily Food intake questionnaire:* This is a daily record of the subject’s eating habits. They will fill it out for 3-days before, during the middle and at the end of the intervention. They will return the 3-days of food records to us once complete.
- *Quality of Life Questionnaire*
- *Laval Questionnaire:* This questionnaire evaluates how obesity has affected the subjects life over the past 4-weeks

***Phase 4: Interventions***

Prior to the intervention subject’s undergoing bariatric surgery will be match paired to receive either standard medical care (SC) or exercise + standard care for 4 weeks. Subject’s not undergoing bariatric surgery will then be randomized to the exercise + diet + standard care group or diet + standard care group for 2 weeks.

*Standard medical care:* If subjects are assigned to this group they will not be provided materials to consume healthy diet and increase exercise participation. Subjects will also be asked to participate in the standard education sessions that are provided to all bariatric surgery patients. This standard care includes meetings with a nutritionist, a psychologist, and a bariatric surgeon.

*Diet + standard medical care*: If subject’s are assigned to this group they will be instructed to follow a weight loss diet plan, created for them by the research team. The diet will restrict your caloric intake to 1000-1400 calories per day. Subject’s will be given two meal replacement shakes each day and advice for preparing a standard, calorie-limited dinner designed for weight loss. Subjects are required to pick up food at least 1 time a week. Subjects will return empty bottles of meal shakes to confirm compliance.

*Exercise training* + *standard medical care for bariatric patients*: If subject’s are assigned to this group they will participate in exercise training and the standard medical care outlined above. Subject’s will be asked to exercise 5 days/week. Subject’s will be able to attend regularly supervised and scheduled training sessions with the exercise physiology staff members in Memorial Gym or they can exercise on their own. Overall, the training program will ask subject’s to exercise 5 times per week and 30 min/day. Subject’s heart rate will be monitored continuously during all training sessions, and they will be asked to exercise at an intensity 65-85% of their previously measured HRmax. Walking will be the main type of exercise. In addition to this training program, subject’s will participate in the standard education sessions that are provided to all bariatric surgery patients.

*Exercise training +* diet + *standard medical care for non-bariatric subjects*: Subject’s assigned to this group will participate in diet program as well as the standard medical care outlined above. For the exercise training, subjects will exercise in Memorial Gymnasium under full supervision by an exercise specialist. The subject will be instructed by an exercise specialist to perform exercise at an exercise intensity that alternates between easy and hard (i.e. interval exercise). They will be instructed to exercise for 60 minutes each session. The first 2 days of training will consist of an introduction to the exercise that includes measures of breath samples to ensure proper exercise intensity and determine how much sugar and fat they are burning for energy. On days 3-7 they will exercise in the select exercise intensity (i.e. interval), and on day 8 they will be given the day off. On days 9-13 they will finish the training program. On day 9 subjects will complete their normal exercise session while the study team collects breath samples to account for caloric expenditure during an exercise bout. This will allow the research team to better understand the caloric deficit of each subject when combining diet with exercise. On day 13, breath samples will be repeated to verify exercise intensity and amount of sugar and fat burned for energy. Heart rate will be measured during all exercise sessions to make sure the intensity is appropriate.

***Phase 5: Post-Intervention Testing***

All study testing visits highlighted above in phase 2 will be repeated.

***Phase 6: Bariatric Surgery (if applicable)***

Subjects undergo bariatric surgery within 1 week, although **3-4 days** following the phase 5 testing will be targeted. Following your surgery, the research team will examine certain details from the procedure. Specifically, the research team will look at the bariatric surgery operation time and any surgery complications that may occur. A fat biopsy will be collected during the time of surgery and within 3-4 days of the intervention assignment.

***Phase 7: Post-Bariatric Surgery Testing (if applicable)***

After bariatric surgery, the subjects will repeat phases 3 and 5 as part of post-operation testing to determine if pre-operative therapy impacts post-surgery metabolic outcomes.

**Study Schedule Visit Overview for Bariatric Patients**

| **Study Visit** | **Screening Visit 1** | **Screening Visit 2^** | **Pre-Test Visit 3** | **Exercise Training 1-2 d/wk***  **Visits 4-11** | **Post-Intervention Testing**  **Visit 4**  **(or Visit 12)^** | **Bariatric Surgery**  **Visit 5**  **(or Visit 13)** | **Post- Bariatric Surgery Testing**  **Visit 6**  **(or Visit 14)^** |
| --- | --- | --- | --- | --- | --- | --- | --- |
| Consent | X |  |  |  |  |  |  |
| Blood Draw | X |  | X |  | X |  | X |
| History and Physical |  | X | X |  | X |  |  |
| Urine Collection (Nitrogen Analysis) |  |  | X |  | X |  | X |
| Stool Collection  (Gut Bacteria) |  |  | X |  | X |  | X |
| VO2peak (Treadmill testing) |  | X |  |  | X |  | X |
| Timed Walk Test (6-MWT) |  | X |  |  | X |  | X |
| Body Fat measurement |  | X |  |  | X |  | X |
| Adipose Biopsy |  |  | X |  |  | X |  |
| MMTT |  |  | X |  | X |  | X |
| Indirect Calorimetry |  |  | X |  | X |  | X |
| Blood Pressure, AI and PWV tests |  |  | X |  | X |  | X |
| Diet Record |  |  | X |  | X |  | X |
| Appetite Questions |  |  | X |  | X |  | X |
| Quality of Life Questions |  |  | X |  | X |  | X |
| Laval Questionnaire |  |  | X |  | X |  | X |
| Accelerometer |  | X |  |  | X |  | X |
| Heart Rate Monitors |  |  |  | X |  |  |  |
| Urine Pregnancy Test (women) |  | X | X |  | X |  | X |

Note: Subjects will be match paired to the group for pre-operative treatment. ^Visit 2 is designed to be performed in 1 day. However, if time does not permit this day may be separated in to 2 visits to accommodate needs. Also, we perform fitness and body fat tests within 3 days post blood work to minimize any time constraint involved in blood work tests. ** Exercise Training will be performed at UVA in Memorial Gymnasium and fully supervised by an exercise physiologist. Heart rate monitors are to be worn when at home exercising as well.

**Study Schedule Visit Overview for Non-Bariatric Subjects**

| **Study Visit** | **Screening Visit 1** | **Screening Visit 2^** | **Pre-Test Visit 3** | **Exercise Training**  **Visits 4-17** | **Diet Pick Up****  **Visits**  **4-5** | **Post-Intervention Testing**  **Visit 18**  **(or Visit 6)** |
| --- | --- | --- | --- | --- | --- | --- |
| Consent | X |  |  |  |  |  |
| Blood Draw | X |  | X |  |  | X |
| History and Physical |  | X | X |  |  | X |
| Urine Collection (Nitrogen & Microparticle Analysis) |  |  | X |  |  | X |
| Stool Collection  (Gut Bacteria) |  |  | X |  |  | X |
| VO2peak (Cycle ergometer testing) |  | X |  |  |  | X |
| Timed Walk Test (6-MWT) |  | X |  |  |  | X |
| Body Fat measurement |  | X |  |  |  | X |
| Adipose Biopsy |  |  | X |  |  | X |
| OGTT |  |  | X |  |  | X |
| Indirect Calorimetry |  |  | X |  |  | X |
| Blood Pressure, FMD, AI, and PWV tests |  |  | X |  |  | X |
| Diet Record |  |  | X |  |  | X |
| Appetite Questions |  |  | X |  |  | X |
| Quality of Life Questions |  |  | X |  |  | X |
| Laval Questionnaire |  |  | x |  |  | x |
| Accelerometer |  | X |  |  |  | X |
| Heart Rate Monitors |  |  |  | X |  |  |
| Meal Shakes |  |  |  |  | X |  |
| Urine Pregnancy Test (women) |  | X | X |  |  | X |

Note: Subjects will be randomized to diet or diet+exercise intervention group. ^Visit 2 is designed to be performed in 1 day. However, if time does not permit this day may be separated in to 2 visits to accommodate needs. Also, we perform fitness and body fat tests within 3 days post blood work to minimize any time constraint involved in blood work tests. ** Exercise Training for subjects not receiving bariatric surgery will be performed at UVA in Memorial Gymnasium and fully supervised by an exercise physiologist. *** Meal Shakes will be provided for the diet. Subjects are asked to pick up these meal shakes 1 time a week and return empty bottles. If subjects are also exercising, diet shakes will be picked up during training sessions.

**3. Will you be using data/specimens in this study that were collected previously, with the use of a research consent form, from another research study?** NO

**4. Will any of the procedures listed in item # 2 have the potential to identify an incidental finding? This includes ALL procedures, assessments and evaluations that are being done for RESEARCH PURPOSES that may or may not be considered investigational.**

There is the possibility that some subjects will meet the ADA diagnostic criteria for type 2 diabetes during screening or the outpatient visits (fasting blood glucose >125 mg/dl or HbA1c ≥6.5%; ADA Standards of Care 2010). In this case, the subject will be notified of the results. A hardcopy of the results will be provided to the subject.

►IF YES, *check one of the following two options:*

__X___The examination(s) utilize(s) the same techniques, equipment, etc., that would be used if the subject were to have the examination(s) performed for clinical care. **There exists the potential for the discovery of clinically significant incidental findings.**

- The PI takes full responsibility for the identification of incidental findings:
- The PI will inform the subjects verbally of all incidental findings that are of clinical significance or are of questionable significance**.**
- A follow-up letter describing the finding should be provided to the subject with instructions to either show the letter to their PC or if the subject has no PCP, the subject should be instructed to make an appointment at UVa or at the Free Clinic**.**

**5. Do any of the procedures listed above, under question # 2, utilize any imaging procedures for RESEARCH PURPOSES?**

**Examples:** ultrasound, CT scans/ x-rays etc.

Answer/Response: No

**6. Will you be using viable embryos?** NO

1. **Will you be using embryonic stem cells?** NO
2. **Are any aspects of the study kept secret from the participants?** NO
3. **Is any deception used in the study?** NO
4. **If this protocol involves study treatment, explain how a subject will be transitioned from study treatment when they have completed their participation in the study.** N/A

# Family History/Pedigree

**1. What kind of information is being sought?**

Whether the subject’s first degree relatives have Type 1 or Type 2 diabetes. Also, if there is a

family history of CVD.

**2. What identifiers will be recorded with any of the following info (e.g. names, initials, relationship such as mother, father, brother, sister, random number)?**

Nothing other than what relationship they have to the subject. No names, initials or anything else that would identify the person will be recorded

**3. Will HIPAA identifiers will recorded?**

From the clinical chemistry lab for screening labs: Name, hx #, age, date

Samples collected during the study itself: Protocol #, Name, History #, date

**IF YES, provide a justification for recording/keeping HIPAA identifiers:**

Clinical chemistry would be linked to MRN for subjects to have.

1. **Does any of the information sought potentially expose the subject or a family member to additional risk?** NO

# Specimens

**Specimen Information**

**1. Describe the type of specimen to be used:** Blood, urine, fecal matter, and adipose tissue

**2. Will the specimen be obtained BEFORE a subject has signed a consent form?** NO

**3. Will you be using discarded specimens?** NO

**►IF NO, where will blood be drawn?** *Check all that apply*

_____in clinical labs

___x_in the clinical research unit (CRU)

_____ in a clinical setting

_____ in a research lab which has an IBC#

_____ Other: Explain Answer/Response:

**►IF NO, who will draw the blood?**

__x__ a member of the study team who is an individual licensed to practice medicine or osteopathy, a nurse practitioner, or a physician assistant employed by UVa School of Medicine

_____a member of the study team who is a person trained to draw blood by an individual licensed to practice medicine or osteopathy, a nurse practitioner, or a physician assistant employed by UVa. *Written documentation of training will be kept in research files. Individual also has current training in handling of blood borne pathogens*

**►IF NO, and taking a blood sample, will blood be taken more than 2 times/week?** NO

**►IF NO, and taking a blood sample, check the option(s) below which match the subject population.**

__X___ healthy, non-pregnant adults who weigh at least 110 pounds.

__X___ Amount will NOT exceed 550 cc in an 8 week period

_____ Amount to exceed 550 cc in an 8 week period

_____ Non- healthy or pregnant adults and/or children

_____ Amount will NOT exceed the lesser of 50ml or 3 ml/kg in an 8 week period

_____ Amount will exceed the lesser of 50 ml or 3 ml/kg in an 8 week period

**4. Will any additional data be linked to the specimen by way of a code?** Yes- -data obtained on this subject for this study

**5. Will the analysis on the specimen be done soon (within 24 hours) after it is collected?**

No, not for the majority of blood collected. For example, blood glucose will be analyzed at the time blood is collected. All remaining blood (e.g. insulin, etc.) and adipose tissue sample will be analyzed together to minimize variance between assays.

**►IF NO, where will the specimen be stored until analysis is done?**

At UVA in a freezer in the core lab of the CRU or the Barrett lab located in Fontaine Research Park 450 Ray C Hunt Drive, Room 1280 (IBC numbers 132-02 and 705-09 respectively) or the McNamara lab located in the Cardiovascular Research Center in MR5, Room G217 and 1203 (IBC number 131-97). Adipose tissue samples may also be stored in the Isakson lab located in MR4, Room 6072 (IBC number 505-07).

**Specimen Shipping**

**1. Do you plan to ship any specimens outside of UVA?** NO

**2. Do you confirm you will have a signed Material Transfer Agreement with Grants and Contract office prior to shipping the specimens?**

N/A

**3. To whom will the specimen be shipped?**

N/A

**4. What will the outside site do with the specimens?**

N/A

**5. What information that will be on the specimen label when the specimen is sent outside of UVa.?**

N/A

**6. Will any additional information be sent with the sample?**

N/A

**►IF, YES, list what will be sent?**

N/A

1. **Will any HIPAA identifiers be sent out with the specimens?**

N/A

**INSTRUCTIONS:**

Unless the sample is going to a CLIA approved outside lab for clinically validated testing, the IRB-HSR will usually NOT approve any HIPAA identifier except dates to accompany the specimen. If other HIPAA identifiers such as name, initials are to be included you must provide a very strong justification for their inclusion.

NOTE to Study Team: personnel performing this function must take the appropriate Department of Transportation training. The School of Medicine Clinical Trials Office has the training available. They can be reached at 924-8530.

Note to IRB Staff: If the information sent outside of UVa with the specimen meets the criteria of “Identifiable” and the study does not have a consent form- the disclosure would require Tracking under HIPAA regulations. If it meets the criteria of a Limited Data Set, a Data Use Agreement will be required.

**INSTRUCTIONS:** Check all HIPAA identifiers below that will be shared outside of UVa.

|  | 1. Name |
| --- | --- |
|  | 2. All geographic subdivisions smaller than a state, including street address, city, county, precinct, zip code, and their equivalent geocodes, except for the initial three digits of the zip code if, according to the current publicly available data from the Bureau of the Census: (1) The geographic unit formed by combining all zip codes with the same 3 initial digits contains more than 20,000 people and (2) The initial 3 digits of a zip code for all such geographic units containing 20,000 is changed to 000. |
|  | 3. All elements of dates (except year) for dates directly related to an individual, including birth date, admission date, discharge date, date of death; and all ages over 89 and all elements of dates (including year) indicative of such age, except that such ages and elements may be aggregated into a single category of age 90 or older.  *[This means you may record the year but not record the month or day of any date related to the subject if the subject is under the age of 89. In addition if the subject is over the age of 89 you may not record their age and you may not record the month, day or year of any date related to the subject ]* |
|  | 4. Telephone numbers |
|  | 5. Fax numbers |
|  | 6. Electronic mail addresses |
|  | 7. Social Security number |
|  | 8. Medical Record number |
|  | 9. Health plan beneficiary numbers |
|  | 10. Account numbers |
|  | 11. Certificate/license numbers |
|  | 12. Vehicle identifiers and serial numbers, including license plate numbers |
|  | 13. Device identifiers and serial numbers |
|  | 14. Web Universal Resource Locators (URLs) |
|  | 15. Internet Protocol (IP) address numbers |
|  | 16. Biometric identifiers, including finger and voice prints |
|  | 17. Full face photographic images and any comparable images |
|  | 1. Any other unique identifying number, characteristic, code that is derived from or related to information about the individual (e.g. initials, last 4 digits of Social Security #, mother’s maiden name, first 3 letters of last name.) |
|  | 1. Any other information that could be used alone or in combination with other information to identify an individual. (*e.g. rare disease, study team or company has access to the health information and a HIPAA identifier or the key to the code*.) |

**►IF, YES, why are the HIPAA identifiers needed?**

**INSTRUCTIONS:** If applicable: may use the following example: Sample is being sent to a CLIA certified lab which requires HIPAA identifiers to confirm the clinically validated result is labeled with the correct patient/subject.

Answer/Response: Samples are not being shipped outside UVA.

# Specimen Banking at UVa

**Information Accompanying Specimens or Data**

**1. What information will be on the label of the specimen?**

Specimens stored for yet to be identified future research will be labeled with a unique identifying code specifying the admission number (i.e. study ID), test, sample type, condition, date and collection time point. No name, subject initials, or medical record # will be included on the specimen.

**2. What information will be "linked to" or will accompany the specimen?**

Only a unique code specific to the study will accompany the specimen. That code will be kept by the PI using a password protected database to link identifiers. Only study personnel will have access to the password.

**3. If the samples are identifiable (**have HIPAA identifiers**) explain why they cannot be coded or de-identified.**

No- samples are coded. (Limited data set included date of collection.)

**Collection and/or Storage of Specimens**

**4. How much material (e.g. blood, tissue) will be collected and how will it be collected?**

A total of approximately 600cc of blood will be collected during the entire study (10 weeks) and tested for:

1. Glucose
2. Lipid Profile
3. Blood chemistries
4. C-peptide
5. Free Fatty Acids
6. Insulin
7. Microparticles (for non-bariatric patients only)
8. Biomarkers of inflammatory (e.g. Immune Cells, MCP-1, Hs-CRP, adiponectin, etc.)
9. Adipose tissue fat metabolism markers of inflammation (e.g. MCP-1, UCP-1, etc.)
10. Gut hormones (e.g. GLP-1, Ghrelin)
11. Pregnancy (in females), blood for screening and urine at each admission.

A total of 100cc of urine will be collected during the entire study (10 weeks) and tested for:

1. Microparticles (for non-bariatric patients) will be analyzed using the same exact technique as blood microparticles (ImageStreamX) as routinely done in our UVa Flow Cytometry Core Lab

A total of 8-16 ounces of fecal matter will be collected during the entire study (10 weeks) and tested for gut microbiota (e.g. Bacteroides/Prevotella,Bifidobacterium genus etc.).

A total of 10 grams of adipose tissue will be collected during the pre-intervention testing visit for:

1. PCR analysis of markers of inflammation (e.g. MCP-1, adiponectin, leptin, etc.)

A total of 10 grams of adipose tissue will be collected at the time of surgery (bariatric surgery patients only) for:

1. PCR analysis of markers of inflammation (e.g. MCP-1, adiponectin, leptin, etc.)
2. Isolation of blood vessels to determine insulin-dependent vasodilation of the blood vessels to assess vascular insulin resistance. This will be used to compare in vitro blood vessel response to the intervention when compared to the whole-body in vivo response (Augmentation index).

**5. Could the loss of confidentiality of the subject's health information potentially have a negative impact decisions of health coverage, employment, insurability or any other benefit, or cause social stigmatization*?*** NO

**6. Who will be responsible for storing the specimens?**

UVA PI, Steven K. Malin

**7. Where will the specimens be stored at UVa?**

At UVA in the freezer in the core lab of the CRU initially upon collection. Thereafter, specimens will be stored in the Barrett lab located in Fontaine Research Park 450 Ray C Hunt Drive, Room 1280 (IBC numbers 132-02 and 705-09 respectively). Urine samples will be stored in the Erdbrugger lab located in Jordan Hall (IBC number 4890-16).

**8. Will another research institution or entity outside of UVa ever have control over the specimens?** NO

**9. Can participants withdraw their specimens or request that they be destroyed?**

Yes- Subjects may have samples withdrawn from the bank that have not already been used for other research

# Data and Safety Monitoring Plan

###### 1. Definition:

**1.1 How will you define adverse events (AE)) for this study?**

Check **all** that apply

__X___An adverse event will be considered any undesirable sign, symptom or medical or psychological condition **even if the event is** **not considered to be related** to the investigational drug/device/intervention. Medical condition/diseases present before starting the investigational drug/intervention will be considered adverse events only if they worsen after starting study treatment/intervention. An adverse event is also any undesirable and unintended effect of research occurring in human subjects as a result of the collection of identifiable private information under the research. Adverse events also include any problems associated with the use of an investigational device that adversely affects the rights, safety or welfare of subject s.

**1.2 How will you define serious adverse events?**

Check **all** that apply

__X___A serious adverse event will be considered any undesirable sign, symptom, or medical condition which is fatal, is life-threatening, requires or prolongs inpatient hospitalization, results in persistent or significant disability/incapacity, constitutes a congenital anomaly or birth defect, is medically significant and which the investigator regards as serious based on appropriate medical judgment. An important medical event is any AE that may not result in death, be life-threatening, or require hospitalization but may be considered an SAE when, based upon appropriate medical judgment, it may jeopardize the patient and may require medical or surgical intervention to prevent one of the outcomes listed in the definitions of SAEs.

**1.3 What is the definition of an unanticipated problem?**

Do not change this answer

An unanticipated problem is any event, experience that meets ALL 3 criteria below:

- Is unexpected in terms of nature, severity or frequency given the research procedures that are described in the protocol-related documents AND in the characteristics of the subject population being studies
- Related or possibly related to participation in research. This means that there is a reasonable possibility that the incident may have been caused by the procedures involved in the research study.
- The incident suggests that the research placed the subject or others at greater risk of harm than was previously known or recognized OR results in actual harm to the subject or others
  1. **What are the definitions of a protocol violation and/or noncompliance?**

Do not change this answer

**A protocol violation is defined as any change, deviation, or departure from the study design or procedures of research project that is NOT approved by the IRB-HSR prior to its initiation or implementation.** Protocol violations may be major or minor violations.

**Noncompliance can be a protocol violation OR deviation from** standard operating procedures, Good Clinical Practices (GCPs), federal, state or local regulation**s.** Noncompliance may be serious or continuing.

Additional Information: see the IRB-HSR website at

<http://www.virginia.edu/vpr/irb/HSR_docs/Forms/Protocol_Violations_%20Enrollment_Exceptions_Instructions.doc>

- 1. **If pregnancy occurs how will this information be managed?**

_____ Adverse Event- will follow adverse event recording and reporting procedures outlined in section 3.

_____ Unanticipated Problems- will follow Unanticipated Problem recording and reporting procedures outlined in section 3.

__X___ Other: Document in research records and subject participation will be discontinued at that time

- 1. **What is the definition of a Protocol Enrollment Exception?**

__X___NA- No outside sponsor

- 1. **What is the definition of a data breach?**

Do not change this answer

A data breach is defined in the HITECH Act (43 USC 17932) as an unauthorized acquisition, access, or use of protected health information (PHI) that compromises the security or privacy of such information.

Additional Information may be found on the IRB-HSR Website: [Data Breach](http://www.virginia.edu/vpr/irb/hsr/data_breach.html)

###### 2. Identified risks and plans to minimize risk

**2.1 What risks are expected due to the intervention in this protocol?**

| **Expected Risks related to study participation.** | **Frequency** |
| --- | --- |
| Ecchymosis, hematoma, venous thrombosis or infection at the I.V. site  Fitness tests may cause anxiety and/or claustrophobia  During FMD, subjects may experience mild discomfort (e.g. mild pain, tingling, and/or numbness) in their forearm and/or hand as a result of the prolonged inflation (5 min.) of the blood pressure cuff required for the procedure. | ____Occurs frequently  ____Occurs infrequently  ___x_Occurs rarely  ____Frequency unknown |
| Reproductive Risks | Minimized due to the requirements of this protocol. |
| Violation of subject’s privacy and confidentiality | Minimized due to the requirements of the privacy plan in this protocol |
| Cardiovascular reactions to exercise, such as fainting, syncope, chest pain, or sudden cardiac death. | Occurs rarely (risk of cardiac events during exercise testing: 3/5,000 tests; risk of death during exercise: ~1/18,000 individuals per year) |

**2.2 List by bullet format a summary of safety tests/procedures/observations to be performed that will minimize risks to participants:**

- A careful history and physical examination to exclude those subjects with a history or current symptoms of cardio-pulmonary conditions as well as renal, liver or metabolic disease.
- InBody will be performed by trained personnel
- CBC with differential to screen out people with low hematocrit or evidence of infection or other illness
- Glucose and HbA1c to assess diabetes status
- Comprehensive Metabolic to evaluate kidney and liver function.
- Pregnancy test in females
- Thorough physical examination and medical history will be performed by a physician prior to study entry, with specific attention to exclude those subjects with a history or current symptoms of cardio-pulmonary conditions.
- Liver, kidney, and hematologic function will be assessed from blood samples using standard clinical tests listed below:

CBC, BUN, and creatinine, tests within normal limits

Liver function tests no greater than 3-fold within normal limits

HCT for women > 36%, Men >38%

- Complications of I.V. cannuale will be minimized by aseptic and skilled nursing technique
- The minimal amount of blood required to measure each lab will be removed.
- Each exercise test will be monitored by Exercise Physiology Core Lab personnel who have current training in CPR and AED use, and all protocols will follow the American College of Sports Medicine Guidelines. An AED and a cell phone available to call the code 12 team if necessary. While a physician is not required to be present during exercise testing in this population (according to AHA/ACC/ACSM guidelines), it is standard CRU practice that the study physician is aware of the test and available by page.
- If a subject develops leg pain during the cycle ergometer exercise, the resistance on the device will be decreased.

**2.3 Under what criteria would an INDIVIDUAL SUBJECT’S study treatment or study participation be stopped or modified**

__X___At subject, PI or sponsor’s request

- If subject has any of the less likely to severe side effects for a particular admission that study will be terminated, and depending on the side effect and extent may be excluded from all admissions.
- Any significant blood loss is prevented by regular visual inspection of the catheter insertion site and removing a catheter and applying pressure if there is obvious extravasation of blood into the tissue.
- These catheters are in place for a brief time (~five hours) and careful aseptic technique is used for catheter placement. With these precautions infections secondary to intravenous lines is extremely uncommon. Should an infection occur, we would treat with oral or intravenous antibiotics or if necessary by local drainage.
- If the subject experiences headache, chest pain, vomiting, flushing, or dizziness
- If the subject experiences hypoglycemia with a blood glucose of 60 or less the study will be stopped. It may be stopped at a higher value at the discretion of the Principal Investigator if there is loss of IV access.
- A woman with a positive pregnancy test will be excluded.
- **Exercise Testing**

The ACC/AHA guideline update for exercise testing endorsed by the ACSM is used in the Exercise Physiology Core Laboratory for all exercise protocols. This document provides guidelines for absolute and relative indications for terminating exercise testing (documentation accompanies this submission)

*Exercise Termination:*

Absolute

1. Drop in systolic blood pressure of >10 mm Hg from baseline blood pressure despite an increase in workload, when accompanied by other evidence of ischemia
2. Moderate to severe angina
3. Increasing nervous system symptoms (e.g. ataxia, dizziness, or near syncope)
4. Signs of poor perfusion (cyanosis or pallor)
5. Technical difficulties monitoring the ECG or systolic blood pressure
6. Subject’s desire to stop
7. Sustained ventricular tachycardia
8. ST elevation (>1.0 mm) in leads without diagnostic Q-waves other than V1 or aVR.

Relative

1. Drop in systolic blood pressure of >10 mm Hg from baseline blood pressure despite an increase in workload, in the absence of other evidence of ischemia.
2. ST or QRS changes such as excessive ST depression (>2mm horizontal or down-sloping ST-segment depression) or marked axis shift
3. Arrhythmias other than sustained ventricular tachycardia, including multifocal PVC’s triplets of PVC’s, supraventricular tachycardia, heart block, or bradyarrhythmias.
4. Fatigue, shortness of breath, wheezing, leg cramps, or claudication
5. Development of bundle-branch block or intraventricular conduction delay that cannot be distinguished from ventricular tachycardia
6. Increasing chest pain
7. Hypertensive response: systolic >250 mm Hg, diastolic >115 mm Hg.
   1. **Under what criteria would THE ENTIRE STUDY need to be stopped.**

__X___Per IRB, PI, DSMB, or sponsor discretion

**2.5 What are the criteria for breaking the blind/mask?**

__X___NA – Not blinded/masked

- 1. **How will subject withdrawals/dropouts be reported to the IRB prior to study completion?**

__X___IRB-HSR continuation status form

###### 3. Adverse Event / Unanticipated Problem Recording and Reporting

**3.1 Will all adverse events, as defined in section 1.1, be collected/recorded?** NO

**►IF NO, what criteria will be used?**

__X___Only adverse events deemed related/possibly related to study

**3.2 How will adverse event data be collected/recorded**?

__X___Spreadsheet: paper or electronic

**3.3. How will AEs be classified/graded?**

_X_ Serious/Not serious

- 1. **What scale will the PI use when evaluating the relatedness of adverse events to the study participation?**

__X___The PI will determine the relationship of adverse events to the study

using the following scale:

Related: AE is clearly related to the intervention

Possibly related: AE may be related to the intervention

Unrelated: AE is clearly not related to intervention

**3.5 When will recording/reporting of adverse events/unanticipated problems begin?**

__X___After subject begins study drug/ device placement/intervention /study-related procedure/specimen collection

**3.6 When will the recording/reporting of adverse events/unanticipated problems end?**

__X___ End of study drug/device/intervention/participation

**3.7 How will Adverse Events, Unanticipated Problems, Protocol Violations and Data Breaches be reported? Complete the table below to answer this question**

| **Type of Event** | **To whom will it be reported:** | Time Frame for Reporting | How reported? |
| --- | --- | --- | --- |
| **Any internal event resulting in death that is deemed DEFINITELY related to (caused by) study participation**  *An internal event is one that occurs in a subject enrolled in a UVa protocol* | IRB-HSR | Within 24 hours | IRB Online and phone call  [www.irb.virginia.edu/](http://www.irb.virginia.edu/) |
| **Internal, Serious, Unexpected adverse event** | IRB-HSR | Within 7 calendar days from the time the study team received knowledge of the event.  *Timeline includes submission of signed hardcopy of AE form.* | IRB Online  [www.irb.virginia.edu/](http://www.irb.virginia.edu/) |
| **Unanticipated Problems** that are not adverse events or protocol violations  This would include a Data Breach. | IRB-HSR | Within 7 calendar days from the time the study team received knowledge of the event. | Unanticipated Problem report form.  [*http://www.virginia.edu/vprgs/irb/HSR_docs/Forms/Reporting_Requirements-Unanticipated_Problems.doc*](http://www.virginia.edu/vprgs/irb/HSR_docs/Forms/Reporting_Requirements-Unanticipated_Problems.doc) *)* |
| **Protocol Violations/Noncompliance**  *The IRB-HSR only requires that MAJOR violation be reported, unless otherwise required by your sponsor, if applicable.*  *.* | IRB-HSR | Within 7 calendar days from the time the study team received knowledge of the event. | Protocol Violation, Noncompliance and Enrollment Exception Reporting Form  [*http://www.virginia.edu/vprgs/irb/hsr_forms.html*](http://www.virginia.edu/vprgs/irb/hsr_forms.html)  *Go to 3^rd^ bullet from the bottom.* |
| **Data Breach** | The UVa Corporate Compliance and Privacy Office  ITC: if breach involves electronic data  Police if breach includes items that are stolen:  Stolen on UVA Grounds  OR  Stolen off UVa Grounds- contact police department of jurisdiction of last known location of PHI | As soon as possible and no later than 24 hours from the time the incident is identified.  As soon as possible and no later than 24 hours from the time the incident is identified.  IMMEDIATELY. | UVa Corporate Compliance and Privacy Office- Phone 924-9741  **ITC:**  [Information Security Incident Reporting procedure](https://policy.itc.virginia.edu/policy/policydisplay?id=IRM-012), <http://www.itc.virginia.edu/security/reporting.html>  UVa Police-Phone- (434) 924-7166 |

**4.** **How will the endpoint data be collected/recorded.** Check all that apply

__X___Source documents

**5. Data and Safety Oversight Responsibility**

**5.1. Who is responsible for overseeing safety data for this study?**

INSTRUCTIONS:

e.g. Who is looking at data in aggregate form to identify trends?

Check all that apply

__X___No additional oversight body other than PI at UVa Skip question 5.2

**5.2. What is the composition of the reviewing body and how is it affiliated with the sponsor?** N/A

**5.3. What items will be included in the aggregate review conducted by the PI?**

Check all that apply.

__X___All adverse events

__X___Unanticipated Problems

__X___Protocol violations/Issues of noncompliance

- 1. **How often will aggregate review occur?**

For additional information on aggregate review see: [www.virginia.edu/vpr/irb/hsr/continuations.html#aggreview](http://www.virginia.edu/vpr/irb/hsr/continuations.html#aggreview)

__X___Annually

**5.5. How often will a report, regarding the outcome of the review by the DSMB/DSMC, be sent to the UVa PI?**

A copy of these reports must be sent to the IRB if applicable as soon as they are received by the PI. Do not wait until the next continuation to submit them to the IRB.

__X___NA- PI is not the overall person overseeing the safety data for this study.

**5.6. How will a report of the information discussed in question 5.4 OR 5.5 be submitted to the IRB?**

__X___Part of IRB-HSR continuation status form

# Payment

**1. Are subjects being reimbursed for travel expenses** (receipts /mileage required)**?** NO

**2. Are subjects compensated for being in this study?** YES

►IF YES, answer the following questions (2a-2d).

**2a. What is the maximum TOTAL compensation to be given over the duration of the protocol?** $150 if non-bariatric subject and $500 if a bariatric patient

**2b. Explain compensation to be given.**

Bariatric subjects will be compensated per completion of phases:

Completion of Phase 1-3: $**100 (or $150 if pre-test fat biopsy performed)**

Completion of Phase 4-5: $**250**

Completion of Phase 7: $**100**

***Total payment***: **$450 (or $500 if fat biopsy performed in phase 3)**

Non-Bariatric subjects will be compensated per completion of phases:

Completion of Phase 1-3: $**50 (or $75 if pre-test fat biopsy performed)**

Completion of Phase 4-5: $**50 (or $75 if post-test biopsy performed)**

***Total payment***: **$100 (or $150 if fat biopsies performed)**

Please note for **Bariatric Surgery**

Subjects are having bariatric surgery and related tests as part of their clinical care. Subjects and/or their insurance company must pay for any tests or care required for your clinical care. In addition, subjects and/or their insurance company may also have to pay for other drugs and treatments that are given to help them control any side effects. Subjects will have to pay for any costs not covered by their health plan. Subjects may be responsible for any co-payments or deductibles.

**2c. Is payment pro-rated?** YES

**2d. Is money paid from UVa or State funds (including grant funds) or will items such as gift cards be distributed through UVa?**

Yes, the Curry School Foundation and School of Medicine

►IF YES, answer the following questions [2d(i)-2d(ii)].

**2d(i). How will the researcher compensate the subjects?**

__X___ Check issued to participant via UVA Oracle or State system

**2d(ii). Which category/ categories best describes the process of compensation?**

Choose one of the following 3 options

__X___ All compensation will be made via check issued to participant via UVA Oracle or State system

The preferred method

# Risk/ Benefit Analysis

1. What are the potential benefits for the participant as well as benefits which may accrue to society in general, as a result of this study?

Upon completion of the study participants will receive a report detailing their individual results (e.g. body composition, fitness, effects of exercise on post prandial hyperglycemia). Society in general will benefit from greater understanding of the importance of exercise and/or diet in insulin resistance and maintaining healthy immune cell profiles in individuals undergoing bariatric surgery who are at high risk for developing Type 2 diabetes and CVD.

**2. Do the anticipated benefits justify asking subjects to undertake the risks?**

Yes, since the health benefits of exercise clearly outweigh the risk involved, particularly in morbid obese subjects who are at an elevated risk of future disease. While the current study is examining the short-term exercise effects, participants may be more empowered to incorporate exercise into their daily lives after successful completion of the testing sessions as well as from greater understanding of the importance of exercise on glucose control and vascular health.

# Bibliography

1. Malin SK and Kashyap SR. Differences in weight loss and gut hormones: Rouen-Y Gastric Bypass and Sleeve Gastrectomy Surgery. Curr Obes Report 2015; [Epub ahead of print].

2. Malin SK, et al. Attenuated improvements in adiponectin and fat loss characterize type 2 diabetes non-remission status after bariatric surgery. DOM 2014; 16:1230-8.

3. Shada A, et al. Aerobic exercise is associated with improved weight loss after laparoscopic adjustable gastric banding. Obes Surg 2013; 23: 608-12.

4. Coen P, et al. Clinical trial demonstrates exercise following bariatric surgery improves insulin sensitivity. J Clin Invest 2015; 125: 248-57.

5. Khanna V, et al. Adults with long-duration type 2 diabetes have blunted glycemic and ß-cell function improvements after bariatric surgery. Obesity 2015; 23: 523-6.

6. Samat A, et al. Ghrelin suppression is associated with weight loss and insulin action following gastric bypass surgery at 12 months in obese adults with type 2 diabetes. DOM, 2013; 15:963-6.

7. Morris-Rosenfeld S, et al. Understanding the role of B cells in atherosclerosis: potential clinical implications. Expert Rev Clin Immunol 2014; 10: 77-89.

8. Mechanick J, et al. Clinical practice guidelines for the perioperative nutritional, metabolic, and nonsurgical support of the bariatric surgery patient--2013 update: cosponsored by American Association of Clinical Endocrinologists, The Obesity Society, and American Society for Metabolic & Bariatric Surgery. Obesity 2013; 21 Suppl 1: S1-27.

9. Church T, et al. Cardiorespiratory fitness and body mass index as predictors of cardiovascular disease mortality among men with diabetes. Arch Intern Med 2005; 165: 2114-20.

10. Bond D, et al. Exercise improves quality of life in bariatric surgery candidates: results from the Bari-Active trial. Obesity (Silver Spring) 2015; 23: 536-42.

11. Malin SK, et al. Exercise Training with Weight Loss and either a High- or Low-Glycemic Index Diet Reduces Metabolic Syndrome Severity in Older Adults. Ann Nutr Metab 2012; 61: 135-141.

12. Malin SK, et al. Insulin sensitivity and metabolic flexibility following exercise training among different obese insulin resistant phenotypes. Am J Physiol Endocrin Metab 2013; 15:E1292-8.

13. Despres JP, Lemieux I, Prud’homme D. Treatment of obesity: need to focus on high risk abdominally obese patients. BMJ 2001;322(7288):716–720.

14. Samuel VT, Shulman GI. Mechanisms for insulin resistance: common threads and missing links. Cell 2012;148(5):852–871.

15. Nakamura K, Fuster JJ, Walsh K. Adipokines: A link between obesity and cardiovascular disease. Journal of Cardiology 2014;63:250-259.

16. Varbo A, Benn M, Smith GD, Timpson NJ, Tybjærg-Hansen A, Nordestgaard BG. Remnant cholesterol, low-density lipoprotein cholesterol, and blood pressure as mediators from obesity to ischemic heart disease. Cir Res 2015;116:665-673.

17. Iantorno M, Campia U, Di Daniele N, Nisticò S, Forleo GB, Cardillo C, Tesauro M. Obesity, inflammation and endothelial dysfunction. Journal of Biological Regulators & Homeostatic Agents 2014;28(2):169-176.

18. Sjöström L. Lindroos AK, Peltonen M, Torgerson J, Bouchard C, Carlsson B, Dahlgren S, Larsson B, Narbro K, Sjöström CD, Sullivan M, Wedel H; Swedish Obese Subjects Study Scientific Group. Lifestyle, diabetes, and cardiovascular risk factors 10 years after bariatric surgery. N Engl J Med 2004;351(26):2683–2693.

19. Pories WJ, Caro JF, Flickinger EG, Meelheim HD, Swanson MS. The control of diabetes mellitus (NIDDM) in the morbidly obese with the Greenville gas- tric bypass. Ann Surg 1987;206(3):316–323.

20. Buchwald H, Avidor Y, Braunwald E, et al. Bariatric surgery: a systematic review and meta-anlysis. JAMA. 2004;292:1724–37.

21. Hall TC, Pellen MGC, Sedman PC, Jain PK. Preoperative factors predicting remission of type 2 diabetes mellitus after Roux-en-Y gastric bypass sugery for obesity. Obes Surg 2010;20:1245-1250.

22. Schauer PR, Burguera B, Ikramuddin S, et al. Effect of laparoscopic roux-en y gastric bypass on type 2 diabetes mellitus. Ann Surg. 2003;238(4):467–85.

23. Torquati A, Lufti R, Abumrad N, et al. Is Roux en Y gastric bypass surgery the most effective treatment for type 2 diabetes mellitus in morbidly obese patients? J Gastrointest Surg. 2005;9 (8):1112–6.

24. Direk K, Cecelja M, Astle W, Chowienczyk P, Spector TD, Falchi M, Andrew T. The relationship between DXA-based and anthropometric measures of visceral abdominal fat and morbidity in women. BMC Cardiovascular Disorders 2013;13(25).

25. DeFronzo RA, Abdul-Ghani MA. Preservation of ß-cell function: the key to diabetes prevention. J Clin Endocrinol Metab 2011; 96: 2354–2366.

26. Malin SK, Finnegan S, Fealy CE, Filion J, Rocco MB, Kirwan JP. Beta-Cell dysfunction is associated with metabolic syndrome severity in adults. Metab Syndr Relat Disord 2013; 12:79–85.

27. Appachi SK, Kashyap SR. ‘Adiposopathy’ and cardiovascular disease: the bene- fits of bariatric surgery. Curr Opin Cardiol 2013; 28: 540–546.

28. Masquio DCL, de Piano A, Sanches PL, Corgosinho FC, Campos RMS, Carnier J, de Silva PL, Caranti DA, Tock L, Oyama LM, Oller do Mascimento CM, de Mello MT, Tufik S, Damaso AR. The effect of weight loss magnitude on pro-/anti-inflammatory adipokines and carotid intima-media thickness in obese adolescents engaged in interdisciplinary weight loss therapy. Clinical Endocrinology 2013;79:55-64.

29. Vlachopoulos C, Aznaouridis K and Stefanadis C. Prediction of cardiovascular events and all-cause mortality with arterial stiffness: a systematic review and meta-analysis. J Am Coll Cardiol 55: 13: 1318-27, 2010.

30. Wascher TC, Schmoelzer I, Wiegratz A, Stuehlinger M, Mueller-Wieland D, Kotzka J, Enderle M. Reduction of postchallenfe hyperglycemia prevents acute endothelial dysfunction in subjects with impaired glucose tolerance. Euro J Clin Invest. 2005;35(9):551-7.

31. Williams SB, Goldfine AB, Timimi FK, Ting HH, Roddy MA, Simonson DC, Creager MA. Acute hyperglycemia attenuates andothelium-dependent vasodilation in humans in vivo. Circulation. 1998;97(17):1695-701.

32. Liu B, Kuang L, Liu J. Bariatric surgery relieves type 2 diabetes and modulates inflammatory factors and coronary endothelium eNOS/iNOS expression in db/db mice. Can J Physiol Pharmacol 2014;92(1):70-77.

33. Samaras, K., et al., Reduced arterial stiffness after weight loss in obese type 2 diabetes and impaired glucose tolerance: the role of immune cell activation and insulin resistance. Diab Vasc Dis Res, 2013. 10(1): p. 40-8.

34. Viardot, A., R.V. Lord, and K. Samaras, The effects of weight loss and gastric banding on the innate and adaptive immune system in type 2 diabetes and prediabetes. J Clin Endocrinol Metab, 2010. 95(6): p. 2845-50.

35. Iancu, M.E., et al., Favorable changes in arterial elasticity, left ventricular mass, and diastolic function after significant weight loss following laparoscopic sleeve gastrectomy in obese individuals. Obes Surg, 2014. 24(3): p. 364-70.

36. Wing RR, Bolin P, Brancati FL, Bray GA, et al. Cardiovascular effects of intensive lifestyle intervention in type
2 diabetes. New Engl J Med 2013;369(2):145–154.

37. Knowler WC, Barrett-Connor E, Fowler SE, Hamman RF, Lachin JM, Walker EA, Nathan DM, Diabetes Prevention Program Research Group. Reduction in the incidence of type 2 diabetes with lifestyle intervention or metformin. N Engl J Med 2002 Feb 7;346(6):393–403.

38. Turnbaugh PJ, Ley RE, Mahowald MA, Magrinia V, Mardis ER, Gordon JI. An obesity-associated gut microbiome with increased capacity for energy harvest. Nature 2006; 444(1027-1031).

39. Ley Re, Turnbaugh PJ, Klein S, Gordon JI. Microbial ecology: Human gut microbes associated with obesity. Nature 2006; 444(1022-1023).

40. Karlsson FH, Fak F, Nookaew I, Tremaroli V, Fagerberg B, Petranovic D, Backhed F, Nielsen J. Symptomatic atherosclerosis is associated with an altered gut metagenome. Nat Comm 2012; 3(1245)

41. Tremaroli V, Karlsson F, Werling M, Stahlman M, Kovtcheva-Datchary P, Olbers T, Fandriks L, le Roux CW, et al. Roux-en-Y Gastric Bypass and Vertical Banded Gastroplasty Induce Long-Term Changes on the Human Gut Microbiome Contributing to Fat Mass Regulation. Cell Metabolism 2015; 22(228-238).

42. Kong LC, Tap J, Aron-Wisnewsky J, Pelloux V, Basdevant A, Bouillot JL, Zucker JD, Dore J, Clement K. Gut microbiota after gastric bypass in human obesity: increased richness and associations of bacterial genera with adipose tissue genes. Am J Clin Nutr 2013;98(15-24).

43. [Yassine HN](http://www.ncbi.nlm.nih.gov/pubmed/?term=Yassine%20HN%5BAuthor%5D&cauthor=true&cauthor_uid=19164269), [Marchetti CM](http://www.ncbi.nlm.nih.gov/pubmed/?term=Marchetti%20CM%5BAuthor%5D&cauthor=true&cauthor_uid=19164269), [Krishnan RK](http://www.ncbi.nlm.nih.gov/pubmed/?term=Krishnan%20RK%5BAuthor%5D&cauthor=true&cauthor_uid=19164269), [Vrobel TR](http://www.ncbi.nlm.nih.gov/pubmed/?term=Vrobel%20TR%5BAuthor%5D&cauthor=true&cauthor_uid=19164269), [Gonzalez F](http://www.ncbi.nlm.nih.gov/pubmed/?term=Gonzalez%20F%5BAuthor%5D&cauthor=true&cauthor_uid=19164269), [Kirwan JP](http://www.ncbi.nlm.nih.gov/pubmed/?term=Kirwan%20JP%5BAuthor%5D&cauthor=true&cauthor_uid=19164269). Effects of exercise and caloric restriction on insulin resistance and cardiometabolic risk factors in older obese adults--a randomized clinical trial. J Gerontol A Biol Sci Med Sci. 2009;64(1):90-5.

# APPENDIX: Legal/Regulatory

**Recruitment**

The following procedures will be followed:

- Finders fees will not be paid to an individual as they are not allowed by UVa Policy.
- All recruitment materials will be approved by the IRB-HSR prior to use. They will be submitted to the IRB after the IRB-HSR has assigned an IRB-HSR # to the protocol.
- Only those individuals listed as personnel on this protocol will recruit and or conduct the consenting process with potential subjects.

**Retention Incentives**

Any item used by the sponsor/ study team to provide incentive to a subject to remain in the study, other than compensation identified in the Payment section, will be submitted to the IRB for review prior to use. The IRB-HSR will provide the study team with a Receipt Acknowledgement for their records. Retention incentive items are such things as water bottles, small tote bags, birthday cards etc. Cash and gift cards are not allowed as retention incentives.

**Clinical Privileges**

The following procedures will be followed:

- Investigators who are members of the clinical staff at the University of Virginia Medical Center must have the appropriate credentials and been granted clinical privileges to perform specific clinical procedures whether those procedures are experimental or standard.
- The IRB cannot grant clinical privileges.
- Performing procedures which are outside the scope of the clinical privileges that have been granted may result in denial of insurance coverage should claims of negligence or malpractice arise.
- Personnel on this protocol will have the appropriate credentials and clinical privileges in place before performing any procedures required by this protocol.
- Contact the Clinical Staff Office- 924-9055 or 924-8778 for further information.

**Sharing of Data/Specimens**

Data and specimens collected under an IRB approved protocol are the property of the University of Virginia. You must have “permission” to share data/ specimens outside of UVa other than for a grant application and or publication. This “permission” may come in the form of a contract with the sponsor or a material transfer agreement (MTA) with others. A contract/ MTA is needed to share the data outside of UVa even if the data includes no HIPAA identifiers and no code that could link the data back to a HIPAA identifier.

- No data will be shared outside of UVa, beyond using data for a grant application and or publication, without a signed contract/MTA approved by the SOM Grants and Contracts office/ OSP or written confirmation that one is not needed.
- No specimens will be shared outside of UVa without a signed contract/MTA approved by the SOM Grants and Contracts office/ OSP or written confirmation that one is not needed.

**Prisoners**

If the original protocol/ IRB application stated that no prisoners would be enrolled in this study and subsequently a subject becomes a prisoner, the study team must notify the IRB immediately. The study team and IRB will need to determine if the subject will remain in the study. If the subject will remain in the study, the protocol will have to be re-reviewed with the input of a prisoner advocate. The prisoner advocate will also have to be involved in the review of future continuations, modifications or any other reporting such as protocol violations or adverse events.

Prisoner- Individuals are prisoners if they are in any kind of penal institution, such as a prison, jail, or juvenile offender facility, and their ability to leave the institution is restricted. Prisoners may be convicted felons, or may be untried persons who are detained pending judicial action, for example, arraignment or trial.
For additional information see the OHRP website at <http://www.hhs.gov/ohrp/policy/populations/index.html>

**Compensation in Case of Injury**

If a subject requests compensation for an injury, the study team should notify the IRB-HSR (924-9634/2439847) the UVa Health System Patient Relations Department (924-8315). As a proactive courtesy, the study team may also notify UVa Health System Patient Safety and Risk Management (924-5595).

On request, the study team should provide the Risk Management Office with the following information/documents:

- Subject Name and Medical Record Number
- Research medical records
- Research consent form
- Adverse event report to IRB
- Any letter from IRB to OHRP

**Subject Complaints**

During a research study, the study team may receive complaints from a subject. If the study team is uncertain how to respond to a complaint, or is unable to resolve it with the subject, the study team may contact the IRB-HSR (924-9634/243-9847), the UVa Health System Patient Relations Department (924-8315).

**Request for Research Records from Search Warrant or Subpoena**

If the study team receives a request for research records from a search warrant or subpoena, they should notify UVa Health Information Services at 924-5136. It is important to notify them if information from the study is protected by a Certificate of Confidentiality.

# APPENDIX: Recruitment

Recruitment includes identifying, review of records to determine eligibility or any contact to determine a potential subjects interest in the study.

*The UVa HIPAA covered entity is composed of the UVa VP Office of Research, the Health System, School of Medicine, School of Nursing, Nutrition Services (Morrisons), the Sheila C. Johnson Center, the Exercise and Sports Injury Laboratory and the Exercise Physiology Laboratory.

1. **How do you plan to identify potential subjects?**

- To "identify" a potential subject refers to steps you plan to take to determine which individuals would qualify to participate in your study. This does NOT include steps to actually contact those individuals.
- If your study involves more than one group of subjects (e.g. controls and cases or subjects and caregivers) note below which groups are being identified by the given method.
- Check the methods you plan to utilize:

a._X___ Chart Review/ Clinic Schedule Review/ Database Review from a database established for health care operations (departmental clinical database) or an Improvement Project (*e.g. Performance Improvement, Practice Improvement, Quality Improvement*).

*If you plan to obtain data from the UVa Enterprise Data Warehouse (EDW) please see option b below.*

DHHS: Study team requests Waiver of Consent to identify potential subjects.

HIPAA: Allowed under Preparatory to Research if PHI to be accessed.

IMPORTANT

Keep in mind that PHI in the medical record may only be accessed by individuals who work under the UVa HIPAA covered entity; which means they meet one of the following criteria:

--a UVa student working in the UVa HIPAA Covered Entity*

--a faculty or staff member in a PAID appointment in the UVA HIPAA Covered Entity*

b__X__ Review of a database that was established to keep data to be used for future research such as the CDR, departmental research database or use of data from a separate current active research protocol.

*If you plan to obtain data from the UVa Enterprise Data Warehouse (EDW) you are required to submit your request to the CDR. The CDR staff will work with the EDW to obtain the data you need.*

DHHS: Study team requests Waiver of Consent to identify potential subjects.

HIPAA: Allowed under Preparatory to Research if PHI to be accessed.

IMPORTANT

Keep in mind that PHI in the medical record may only be accessed by individuals who work under the UVa HIPAA covered entity; which means they who meet one of the following criteria:

--a UVa student working in the UVa HIPAA Covered Entity*

--a faculty or staff member in a PAID appointment in the UVA HIPAA Covered Entity*

The information from which you are obtaining potential subjects must also have an IRB protocol approval. If this item is checked, enter the IRB # below.

**IRB# 17822**

If obtaining information from the Clinical Data Repository (CDR) insert IRB # 10797

1. ____ Patients UVa health care provider supplies the UVa study team with the patients contact information without patients’ knowledge.

DHHS: Study team requests Waiver of Consent to identify potential subjects.

HIPAA: Allowed under Preparatory to Research if PHI will be shared by the health care provider.

IMPORTANT

Keep in mind that PHI may only be given to individuals who work under the UVa HIPAA covered entity; which means they meet one of the following criteria:

--a UVa student working in the UVa HIPAA Covered Entity*

--a faculty or staff member in a PAID appointment in the UVA HIPAA Covered Entity*

1. ____ Patient obtains information about the study from their health care provider. The patient contacts the study team if interested in participating. (Health care provider may or may not also be the a member of the study team)

DHHS: NA

HIPAA: Allowed under Health Care Operations

If this choice is checked, check 3d-INDIRECT CONTACT below.

1. _X___ Potential subjects will not be directly identified. They will respond to an advertisement such as a flyer, brochure etc.

If this choice is checked, check 3d- INDIRECT CONTACT below.

DHHS & HIPAA: NA

1. _____ Potential subjects have previously signed a consent to have their name in a registry/database to be contacted for future studies of this type.

**IRB# of registry/ database:** ________________

DHHS & HIPAA: NA

1. ____ Other*:* Specify Answer/Response:

**If item # a, b or c is checked above and if this protocol involves the use of protected health information do you confirm the following to be true?** N/A

- The use or disclosure is sought solely to review protected health information as necessary to prepare the research protocol or other similar preparatory purposes.
- No PHI will be removed from the UVa covered entity.
- The PHI that the researcher seeks to use or access is necessary for the research purposes.

Answer/Response: N/A

1. **How will potential subjects be contacted?**

To "contact" a potential subjects refers to the initial contact you plan to take to reach a potential subject to determine if they would be interested in participating in your study. This may include direct contact by such methods as by letter, phone, email or in-person or indirect contact such as the use of flyers, radio ads etc.

If your study involves more than one group of subjects (e.g. controls and cases or subjects and caregivers) note below which groups are being contacted by the given method.

Check the methods below you plan to utilize:

a.__X__Direct contact of potential subjects by the study team via letter, phone, direct e-mail. Members of study team ARE NOT health care providers of patients. Information will not be collected from psychotherapy notes.

Note: Letter, phone, direct email scripts must be approved by IRB prior to use. See [IRB-HSR Website](http://www.virginia.edu/vpr/irb/hsr/advertising.html) for templates.

DHHS/HIPAA: Study team requests a Waiver of Consent and Waiver of HIPAA Authorization to contact potential subjects.

IMPORTANT:

Keep in mind that if PHI was collected during the identification phase that contact with potential subjects may only be performed by individuals who work under the UVa HIPAA covered entity; which means they meet one of the following criteria:

- a UVa student working in the UVa HIPAA Covered Entity*
- a faculty or staff member in a PAID appointment in the UVA HIPAA Covered Entity*

b.____Potential subjects will be approached while at UVa Hospital or Health Clinic by a person who is NOT a member of their health care team. Information will not be collected from psychotherapy notes.

DHHS & HIPAA: Study team requests a Waiver of Consent and a Waiver of HIPAA Authorization to contact potential subjects.

IMPORTANT:

Keep in mind that contacting individuals in a clinical setting may only be performed by individuals who work under the UVa HIPAA covered entity; which means they meet one of the following criteria:

a UVa student working in the UVa HIPAA Covered Entity*

a faculty or staff member in a PAID appointment in the UVA HIPAA Covered Entity*

You should share the following information with the potential subject:

- Your name
- Who you are: physician, nurse etc. at the University of Virginia.
- Why you want to speak with them
- Ask if you have their permission to explain the study to them
- If asked about how you obtained their information use one of the following as an option for response.
  - DO NOT USE THIS RESPONSE UNLESS YOU HAVE OBTAINED PERMISSION FROM THEIR UVa PHYSICIAN: Your doctor, Dr. insert name wanted you to be aware of this research study and gave us permission to contact you.
  - We obtained your information from your medical records at UVa.
  - Federal regulations allow the UVa Health System to release your information to researchers at UVa, so that we may contact you regarding studies you may be interested in participating. We want to assure you that we will keep your information confidential.
- IF THE PERSON SEEMS ANGRY, HESITANT OR UPSET, THANK THEM FOR THEIR TIME AND DO NOT ENROLL THEM IN THE STUDY. YOU MAY ALSO REFER THEM TO THE IRB-HSR AT 924-9634.

c.____Direct contact of potential subjects by the study team by approaching in person at UVa or via letter, phone, direct e-mail. Members of study team contacting potential subjects ARE health care providers of patients.

If you are not approaching them in person but using a letter, phone call or direct email please note that the letter, phone, direct email scripts must be approved by IRB prior to use.

See [IRB-HSR Website](http://www.virginia.edu/vpr/irb/hsr/advertising.html) for templates.

DHHS: Study team requests a Waiver of Consent to contact potential subjects

HIPAA: Allowed under Health Care Operations.

d.__X__ Indirect contact (flyer, brochure, TV, broadcast emails, patient provided info about the study from their health care provider and either the patient contacts study team or gives their healthcare provider permission for the study team to contact them.)

The indirect method used (flyer, brochure, TV, broadcast emails) must be approved by the IRB prior to use. The IRB does not need to review any type of script to use when the potential subject responds to the indirect method.

DHHS & HIPAA: NA

1. ____ Potential subjects are not patients. The study does not include obtaining subjects health information. Subjects will be contacted directly via email, phone, letter or presentation in group setting with consent then obtained individually in a private setting.

If you are not approaching them in person but using a letter, phone call or direct email please note that the letter, phone, direct email scripts must be approved by IRB prior to use.

See [IRB-HSR Website](http://www.virginia.edu/vpr/irb/hsr/advertising.html) for templates.

DHHS: Study team requests a Waiver of Consent to contact potential subjects.

HIPPA: NA

1. **Will any additional information be obtained from a potential subject during "prescreening"?**

Yes. The list of prescreening questions will be asked as follows:

1. Do you smoke, or have you smoked in the last 6 months?
2. What is your weight and height?
3. Do you have normal mensuration?
4. Have you ever been diagnosed with heart or blood vessel disease?
5. How much physical activity do you perform routinely?
6. Have you ever been told by a medical doctor not to exercise?
7. Have you lost or gained weight in the last 3 months?
8. Do you have any current or chronic medical problems?
9. Do you have any allergies?
10. What is your date of birth?
11. Are you able and willing to participate in any treatment group?
12. Are you currently taking any medications?
13. Name, address, phone number, email-we only need this to book their screening appointment if they pass prescreening

IF YES, submit any documents that will be used to collect pre-screening information so that the IRB may confirm what questions will be asked.

NOTE: To comply with HIPAA regulations only the minimum necessary information may be collected at this time. This means that only questions pertaining to the Inclusion and Exclusion Criteria may be asked.

IF YES,

DHHS: study team requests a Waiver of Documentation of Consent for Pre-screening questions.

HIPPA:

HIPAA does not apply if:

--no PHI is collected or

--if PHI is collected from a potential subject by an individual from a department that is not part of the HIPAA covered entity.

HIPAA does apply if the collection occurs by individuals* who work in a department that is part of the HIPAA covered entity.

In this case the collection will be covered under Health Care Operations/

These individuals are those that meet one of the following criteria:

--a UVa student working in the UVa HIPAA Covered Entity*

--a faculty or staff member in a PAID appointment in the UVA HIPAA Covered Entity*

**IF YES, Will any of the questions involve health information?** Yes

**IF YES, will you collect HIPAA identifiers with the health information? Yes**

**IF YES, which HIPAA identifiers will be recorded?**

Name, DOB, Address and phone number, email. These are needed to book the screening visit.

**Do you confirm that health information with HIPAA identifiers will not be shared outside of UVa until a consent form is signed or only shared in a de-identified manner?** YES

1. **Do you plan to ask the subjects to do anything, other than answering questions, for the study prior to signing a consent?** YES

For example: come to the first visit fasting, stop taking medications that may be an exclusion criteria, change diet. As this is still part of pre-screening one is not allowed to gather information that is not directly related to inclusion/exclusion criteria or other issues of suitability (e.g. is person able to come to UVa for multiple visits)

NOTE:

Only those members of the study team with a DEA# (license to prescribe drugs) are allowed to determine if a potential subject may be asked/informed to stop taking a drug which is an exclusion criteria.

It is recommended that the potential subject notify their health care provider if they plan to stop a prescription drug.

**►IF YES, explain in detail what you will ask them to do**.

They will be asked to fast after midnight for the screening blood draw that will be done after signing the consent form the following morning.

Tips to Study Team

You must document their verbal consent in the study records.

If a subject is asked to stop taking a drug, document the date and name of the person on the study team giving the verbal order to stop medications (again- must be a person with a DEA#).

DHHS: Study team requests the use of Verbal Consent (Waiver of Documentation of Consent) for minimal risk screening procedures.

HIPPA:

If the individual, obtaining consent, works under the HIPAA Covered Entity this is covered under Health Care Operations

If the individual obtaining consent does not work under the HIPAA covered entity, HIPAA does not apply.

1. **How will the consenting process take place with either the prospective subject, the subject’s legally authorized representative or parent/legal guardian of a minor ( if applicable)?**

HIPPA:

If the individual, obtaining consent, works under the HIPAA Covered Entity consenting is covered under Health Care Operations.

If the individual obtaining consent does not work under the HIPAA covered entity, HIPAA does not apply.

Describe the setting for the consent.

Who will discuss the study with the potential subject?

How will you assess subject understanding?

How much time will pass between obtaining written consent and initiation of study procedures?

See Protocol Examples: [Consenting Process](http://www.virginia.edu/vpr/irb/HSR_docs/How_will_consenting_process_take_place.doc) for examples of how to answer this question.

If recruiting minors, specify how parental /guardian consent will be obtained prior to approaching the minor.

At first contact the person obtaining consent will explain the consent form verbally, asking open ended questions to determine if the potential subject understands what is being covered in the consent form. Questions might include:

- Would you summarize for me what you believe will be done to you if you are in this study?
- Would you benefit from this study?
- What do you feel are the risks of being in this study?

Potential subjects will be given an opportunity to ask questions. Their level of understanding will dictate how much time will be spent covering each item. Once all of their questions have been answered, if they decide to participate, they will be asked the pre-screening questions. If the subject meets the pre-screening requirements and they are interested in participating he/she will be sent a copy of the consent form either by email or postal mail, along with instructions on how to contact the coordinator for a screening appointment once the consent has been read.

Questions may be asked and answered over email or the phone. If the potential subject decides to proceed with the study a screening visit will be scheduled. The person obtaining consent will sign the form and subjects will be given a copy of the signed consent form. Study procedures will then begin. The informed consent process for each individual subject will be documented in the subject’s medical record.

**6. Will subjects sign a consent form for any part of the study?** YES

**7. Will the study procedures be started the same day the subject is recruited for the study?** NO

**8. Is there the potential to recruit economically or educationally disadvantaged subjects, or other vulnerable subjects such as students or employees?**

Yes, it is possible that employees, students or economically disadvantaged subjects will be recruited but only if they respond to the ad.

**IF YES, what protections are in place to protect the rights and welfare of these subjects so that any possible coercion or undue influence is eliminated?**

We will not seek them out

**9. Do you need to perform a “dry run” of any procedure outlined in this protocol?**

NO

# APPENDIX: Privacy Plan for Studies with Consent/HIPAA Authorization

1. **Answer the questions below to describe the plan to protect the data from improper use and disclosure.**

**1A. Will any HIPPA identifiers be collected or received by the UVa study team?**

**INSTRUCTIONS:**

Answer YES to any item below that will be written down/kept/recorded in any way.

If you answer NO to all items it means you would never be able to go back and obtain any additional data about an individual.

| **YES** | **NO** | **HIPAA Identifier** |
| --- | --- | --- |
| **X** |  | 1. Name |
| **X** |  | 2. Postal address information, other than town or city, state, and zip code |
|  | **X** | 3. Age or Date of Birth if over the age of 89 |
| **X** |  | 4. Telephone numbers |
|  | **X** | 5. Fax numbers |
| **X** |  | 6. Electronic mail addresses |
|  | **X** | 7. Social Security number |
| **X** |  | 8. Medical Record number |
|  | **X** | 9. Health plan beneficiary numbers |
|  | **X** | 10. Account numbers |
|  | **X** | 11. Certificate/license numbers |
|  | **X** | 12. Vehicle identifiers and serial numbers, including license plate numbers |
|  | **X** | 13. Device identifiers and serial numbers |
|  | **X** | 14. Web Universal Resource Locators (URLs) |
|  | **X** | 15. Internet Protocol (IP) address numbers |
|  | **X** | 16. Biometric identifiers, including finger and voice prints |
|  | **X** | 17. Full face photographic images and any comparable images |
|  | **X** | 18. Any other unique identifying number, characteristic, code that is derived from or related to information about the individual (e.g. initials, last 4 digits of Social Security #, mother’s maiden name, first 3 letters of last name.) |
|  | **X** | 19. Any other information that could be used alone or in combination with other information to identify an individual.  (*e.g. rare disease, study team or company has access to the health information and a HIPAA identifier or the key to the code*)  Check this item if the key to the code (subject # 1= John Smith) will be kept with the data. |

**INSTRUCTIONS:**

If you did not answer YES to any HIPAA Identifier under 1A) skip to 1E.

No review by ISPRO is required.

**1A(1) Check all locations below where the data with these HIPAA identifiers from Table 1A will be collected or stored together in the same location ( e.g. on the same electronic drive or in the same paper file)**

_____ with specimens-  *If checked list HIPAA identifiers:_____*

**►If HIPAA identifiers stored with the specimens, the following security precautions will be implemented:**

- - - - - Specimens will be kept in a locked freezer/ or locked room

**INSTRUCTIONS:** Choose only one of the following options:

_____ Specimens will be stored with HIPAA identifiers. Access to the freezer/room will be limited to authorized personnel. Specimens with HIPAA identifiers will never be shared outside of UVa without the written permission of the subject.

__X___ Specimens will be stored with a code and no HIPAA identifiers. The key to the code will be kept in a different location than the specimens.

___X__ in paper file with the data- *If checked list HIPAA identifiers: name, initials, birth date, phone number/address.*

*Remember: Initials are considered a HIPAA identifier!*

**►If HIPAA identifiers are stored in a paper file, where will the paper files be stored?**

_____ case report forms will be stored in a *secure area with limited access.*

___x__ questionnaires/surveys will be stored in a *secure area with limited access.*

_____ other *-* Specify Answer/Response:

**NOTE:** "*in a secure area with limited access*" means in a locked cabinet in a locked office or in a locked building in a locked office when unattended and access to the data is limited to study personnel only.

_____ in an electronic file *( HIPAA identifiers will be noted in 1D*. )

**1B. How will data be collected?**

1B(1)._____ Collection of data *ONTO** an individual‑use device (e.g. desktop computer, smart phone app, tablet, laptop)

**INSTRUCTIONS:** *ONTO means the data will reside on the device.

Do not check this box if the device will simply be used to access a server.

*If checked answer the following questions:*

- What kind of device is it (e.g. laptop, tablet, desktop computer)? _____
- Who manages / supports the device (e.g., Health Systems Computing Services (HS/CS), local computer support partner (LSP), self)? _____

**INSTRUCTIONS:** If the device is managed/support by *self* you must follow both the setup and maintenance security standards described on the UVa Office of Information Security, Policy & Records Office (ISPRO) webpage: <http://www.virginia.edu/informationsecurity/device-requirements.html>

- How long with the data remain on the device before it is transferred elsewhere? _____
- Will anyone other than study team members have access to the data on the device? _____
- If yes, please describe: ___________________________________________
- Will data be transferred elsewhere in an **encrypted** secure manner such as the use of SFTP or HTTPS? _____
- If yes, please describe: ___________________________________________
- Are any backups made of the information on the device? _____
- After information is transferred off the device will you **securely** delete all UVa subject data from the device? _____

**INSTRUCTIONS:** For computers not using Windows 8 or newer, download and use the [Secure Delete Program](http://its.virginia.edu/software/displayPackages.php?tId=56) from ITS. If using Windows 8 or newer, click on Secure Delete when deleting a file. For Macintosh computers, select "**Secure Empty Trash**" from the Finder menu**.**

- Does the owner of the device (e.g. phone service provider/ app developer) have any rights to use or access the data either individually or in aggregate? _____

1B(2.)_____ Collection of data via web-based format (e.g. online consent, online surveys) via a non‑UVa website (e.g. NOT HS/CS, ITS)

*See 1B(6) below for an exception.*

*If checked answer the following questions:*

- Provide the web address (URL): _____

**INSTRUCTIONS :** (e.g., https://name1.name2.org/mystudy/login.html)

The URL is in the address bar of your web browser (e.g., Internet Explorer (IE), Firefox, Chrome)

If you need additional assistance contact your department computer support or system administrator for assistance in answering this question.

- How long will the data remain on the non‑UVa website before it is transferred to UVa server or storage?_____
- Will anyone other than study team members have access to the data on the non‑UVa secure server? _____
- Will data be downloaded to a UVa secure server in an encrypted secure manner such as the use of SFTP or HTTPS? _____
- If yes, please describe: _______________________________

*If checked please provide the location:* _____

**INSTRUCTIONS :** (e.g., https://name.hsc.virginia.edu) or \\hscs-ss1\

You may locate the server name behind the drive designator by taking the following step:

*In Windows under Computer, right click on the Drive icon (e.g.,F:). Then click on Properties. The server name and path will appear at the very top of the box.*

If you need additional assistance contact your department computer support or system administrator for assistance in answering this question.

- Are any backups made of the information on the non‑UVa secure server? _____
- After information is transferred elsewhere will you **securely** delete all UVa subject data from the Non‑UVa website? _____
- Do the owners of the non‑UVa website have any rights to use or access the data either individually or in aggregate? _____
- Is there a Business Associates Agreement (BAA) with the provider of the Non- UVa website? _____

1B(3)._____ Directly to a server managed by the principal investigator’s department or school

*If checked, please provide the name of the server*: _____

*DO NOT CHECK this option if data will be collected directly onto School of Nursing SECURE NET or Cancer Center ONCORE systems or an HS/CS server.*

**INSTRUCTIONS :**

You may locate the server name and path by taking the following step:

*In Windows under Computer, right click on the Drive icon (e.g, F). Then click on Properties. The server name and path will appear at the very top of the box.*

If you need additional assistance contact your department computer support or system administrator for assistance in answering this question.

(e.g. name.virginia.edu or \\home2.virginia.edu\research1 )

- What kind of individual-use device will be used to connect to this server?

(e.g. laptop, tablet, desktop computer)? __ ___

- Who manages / supports this individual-use device (e.g., Health Systems Computing Services (HS/CS), local computer support person (LSP), self)? _____
- *If checked please list how to contact this support:* __________________

**INSTRUCTIONS:** If the device is managed/support by *self* you must follow both the setup and maintenance security standards described on the UVa Office of Information Security, Policy & Records Office (ISPRO) webpage: http://www.virginia.edu/informationsecurity/device-requirements.html

1B(4)._____ Directly to an Information Technology Services (ITS) managed server.

*If checked, please provide the name of the server:* _____

*DO NOT CHECK this option if data will be collected directly onto School of Nursing SECURE NET or Cancer Center ONCORE systems*.

**INSTRUCTIONS :** (e.g., name.its.virginia.edu or \\home2.virginia.edu\research1 ))

You may locate this server name and path by taking the following step:

*In Windows under Computer, right click on the Drive. Then click on Properties. The URL name will appear at the very top of the box.*

If you need additional assistance contact your department computer support or system administrator for assistance in answering this question.

- What kind of individual-use device will be used to connect to this server? (e.g. laptop, tablet, desktop computer)? __ ___
- Who manages / supports this individual-use device (e.g., Health Systems Computing Services (HS/CS),local computer support person (LSP), self)? _____
- *If checked please list how to contact this support:* __________________

**INSTRUCTIONS:** If the device is managed/support by *self* you must follow both the setup and maintenance security standards described on the UVa Office of Information Security, Policy & Records Office (ISPRO) webpage: http://www.virginia.edu/informationsecurity/device-requirements.html

1B(5).__X__ Directly to a Health Systems Computing Services (HS/CS), School of Nursing SECUREnet with I Key managed server or the Cancer Center ONCORE system that is configured to store data regulated by HIPAA.

*If checked, please provide the name of the server*: ___ HSCS- Udrive

**gcrcserver.hscs.virginia.edu\users**

- What kind of individual-use device will be used to connect to this server? (e.g. laptop, tablet, desktop computer)? Encrypted Laptop/desktop
- Who manages / supports this individual-use device (e.g., Health Systems Computing Services (HS/CS), local computer support person (LSP), departmental technology support group, self)? Self, Computing Services.
- *If checked please list how to contact this support:* Tim Tolsen

**INSTRUCTIONS:** If the device is managed/support by *self* you must follow both the setup and maintenance security standards described on the UVa Office of Information Security, Policy & Records Office (ISPRO) webpage: http://www.virginia.edu/informationsecurity/device-requirements.html

1B(6)._____ Directly to a server managed by the sponsor or CRO in which the data will be sent and stored in an encrypted fashion (e.g. must be shared and stored via Secure FX, Secure FTP, HTTPS, PGP) and the server is configured to store data regulated by HIPAA.

- What kind of individual-use device will be used to connect to this server?

(e.g. laptop, tablet, desktop computer)? __ ___

- Who manages / supports this individual-use device (e.g., Health Systems Computing Services (HS/CS), local computer support person (LSP), departmental technology support group,, self)? _____________________________________
- *If checked please list how to contact this support:* __________________

**INSTRUCTIONS:** If the device is managed/support by *self* you must follow both the setup and maintenance security standards described on the UVa Office of Information Security, Policy & Records Office (ISPRO) webpage: http://www.virginia.edu/informationsecurity/device-requirements.html

1.B(7)._X____ Paper

**►IMPORTANT: If you checked any of the items 1B(1) through 1B(4) submit ISPRO approval with new protocol submission.**

You should consult with ISPRO during the development phase of this protocol if your protocol will involve highly technical issues such as the creation of a website to collect data, software application development, the use of a smart phone app, or if you plan to store identifiable data ONTO a tablet/laptop.

Otherwise submit the protocol to ISPRO for review after the protocol is written.

ISPRO CONTACT INFORMATION:

UVa Office of Information Security, Policy & Records Office (ISPRO)

[www.virginia.edu/ispro](http://www.virginia.edu/ispro)

Email: IT-Security@Virginia.edu

**1C. Will any of the data be stored electronically?**

YES

**1C(1)►IF YES, will it include storage of any health information or other sensitive data?**

Answer/Response: Metabolic and vascular data will be stored, however, all data will be de-identified. Data will be paired with a unique code and the PI will have access to this code.

**1C(2)►IF YES, will you store/keep any of the HIPAA identifiers listed below in electronic format?**

| **YES** | **NO** | **HIPAA Identifier** |
| --- | --- | --- |
|  | **X** | 1. Name |
|  | **X** | 2. Postal address information, other than town or city, state, and zip code ( e.g. street name or GPS) |
|  | **X** | 3. Age if over the age of 89 OR Date of Birth if over the age of 89 |
|  | **X** | 4. Telephone numbers |
|  | **X** | 5. Fax numbers |
|  | **X** | 6. Electronic mail addresses |
|  | **X** | 7. Social Security number |
|  | **X** | 8. Medical Record number |
|  | **X** | 9. Health plan beneficiary numbers |
|  | **X** | 10. Account numbers |
|  | **X** | 11. Certificate/license numbers |
|  | **X** | 12. Vehicle identifiers and serial numbers, including license plate numbers |
|  | **X** | 13. Device identifiers and serial numbers |
|  | **X** | 14. Web Universal Resource Locators (URLs) |
|  | **X** | 15. Internet Protocol (IP) address numbers |
|  | **X** | 16. Biometric identifiers, including finger and voice prints |
|  | **X** | 17. Full face photographic images and any comparable images |
|  | **X** | 18. Any other unique identifying number, characteristic, code that is derived from or related to information about the individual (e.g. initials, last 4 digits of Social Security #, mother’s maiden name, first 3 letters of last name.) |
|  | **X** | 19. Any other information that could be used alone or in combination with other information to identify an individual.  (*e.g. rare disease, study team or company has access to the health information and a HIPAA identifier or a subject-code* ) |

**1C(3)►If you checked any item above, list the HIPAA identifiers that will be kept with the data in the same location (e.g. on the same electronic drive or in the same file).**

**INSTRUCTIONS : If you checked # 19 above:** If the key to the code ( subject # 1= John Smith) will be kept in the same electronic location as the data, list item # 19 below.

Answer/Response: NO

**1D. If you listed any HIPAA identifier under 1C(3), where will the data be stored?**

X NA- No HIPAA identifiers will be stored with the data

1D(1)_____ a server managed by the principal investigator’s department or school that is configured to store data regulated by HIPAA or highly sensitive data.

- If checked, please provide the name of the server: CRU/ U-Drive
- *DO NOT CHECK this option if data will be collected directly onto School of Nursing SECURE NET or Cancer Center ONCORE systems*.

**INSTRUCTIONS :** (e.g.,dept. name.virginia.edu or \\home2.virginia.edu\dept\mst3k)

You may locate the server name and path by taking the following step:

*In Windows under Computer, right click on the Drive icon (e.g., O:). Then click on Properties. The server name and path will appear at the very top of the box.*

If you need additional assistance contact your department computer support or system administrator for assistance in answering this question.

- Contact information for the person(s) who manages / supports this server. _____

SKIP the following two questions if you checked 1B(3)

- What kind of individual-use device will be used to connect to this server? (e.g. laptop, tablet, desktop computer)? _____
- Who manages / supports this individual-use device (e.g., Health Systems Computing Services (HS/CS), local computer support person (LSP), departmental technology group, self)? _____
  - *If checked please list how to contact this support:* __________________
- **INSTRUCTIONS:** If the device is managed/support by *self* you must follow both the setup and maintenance security standards described on the UVa Office of Information Security, Policy & Records Office (ISPRO) webpage: http://www.virginia.edu/informationsecurity/device-requirements.html

1D(2)_____ an Information Technology Services (ITS) managed server that is configured to store data regulated by HIPAA

*DO NOT CHECK this option if data will be collected directly onto School of Nursing SECURE NET or Cancer Center ONCORE systems*.

- If checked, please provide the name of the server: _____

**INSTRUCTIONS :** (e.g., name.its.virginia.edu or \\home2.virginia.edu\dept\mst3k))

You may locate the server name and path by taking the following step:

*In Windows under Computer, right click on the Drive icon (e.g., F:). Then click on Properties. The server name and path will appear at the very top of the box.*

If you need additional assistance contact your department computer support or system administrator for assistance in answering this question.

SKIP the following two questions if you checked 1B(4)

- What kind of individual-use device will be used to connect to this server? (e.g. laptop, tablet, desktop computer)? _____
- Who manages / supports this individual-use device (e.g., Health Systems Computing Services (HS/CS), local computer support person (LSP), departmental technology group,, self)? _____
  - *If checked please list how to contact this support:* __________________

**INSTRUCTIONS:** If the device is managed/support by *self* you must follow both the setup and maintenance security standards described on the UVa Office of Information Security, Policy & Records Office (ISPRO) webpage: http://www.virginia.edu/informationsecurity/device-requirements.html

1D(3)_____ Cloud (UVaBox, UVa-Collab)

- If checked, please provide the name of the server: _____

**INSTRUCTIONS:** Not allowed if you have answered YES to any HIPAA identifier except the use of a code.

**NOTE:** No research data may be stored in a non‑UVa licensed cloud provider such as Dropbox, Google Drive, SkyDrive, Survey Monkey etc.

- What kind of individual-use device will be used to connect to this service? (e.g. laptop, tablet, desktop computer)? _____
- Who manages / supports this individual-use device (e.g., Health Systems Computing Services (HS/CS), local computer support person (LSP), self)? _____
  - *If checked please list how to contact this support:* __________________

**INSTRUCTIONS:** If the device is managed/support by *self* you must follow both the setup and maintenance security standards described on the UVa Office of Information Security, Policy & Records Office (ISPRO) webpage: http://www.virginia.edu/informationsecurity/device-requirements.html

1D(4)_____ Directly to a Health Systems Computing Services (HS/CS), School of Nursing SECUREnet with I Key managed server or the Cancer Center ONCORE system that is configured to store data regulated by HIPAA.

*If checked, please provide the name of the server*: _____

**INSTRUCTIONS :** (e.g., \\hscs\name.virginia.edu or \\hscs\share-1\)

You may locate the server name and path by taking the following step:

*In Windows under Computer, right click on the Drive icon (e.g., F:). Then click on Properties.*

*The server name and path will appear at the very top of the box.*

If you need additional assistance contact your department computer support or system administrator for assistance in answering this question.

SKIP the following two questions if you checked 1B(5)

- What kind of individual-use device will be used to connect to this server? (e.g. laptop, tablet, desktop computer)? Encrypted Laptop/desktop
- Who manages / supports this individual-use device (e.g., Health Systems Computing Services (HS/CS), local computer support person (LSP), departmental technology group self)? _____
  - *If checked please list how to contact this support:* Tim Tolsen

**INSTRUCTIONS:** If the device is managed/support by *self* you must follow both the setup and maintenance security standards described on the UVa Office of Information Security, Policy & Records Office (ISPRO) webpage: http://www.virginia.edu/informationsecurity/device-requirements.html

1D(5)_____ a server managed by the sponsor or CRO in which the data will be sent and stored in an encrypted fashion (e.g. must be shared and stored via Secure FX, Secure FTP, HTTPS, PGP) onto a server that is configured to store data regulated by HIPAA.

**INSTRUCTIONS:** The study team should confirm the security of the site with the sponsor, CRO or other outside group.

*NOT ALLOWED if you have answered YES to any HIPAA identifier above and data will not be sent/stored in an encrypted manner.*

**►IMPORTANT: If you checked any of the items 1D(1) - 1D(3), submit ISPRO approval with new protocol submission.**

You should consult with ISPRO during the development phase of this protocol if your protocol will involve highly technical issues such as the creation of a website to collect data, software application development, the use of a smart phone app, or if you plan to store identifiable data ONTO a tablet/laptop. Otherwise submit the protocol to ISPRO for review after the protocol is written.

ISPRO CONTACT INFORMATION:

UVa Office of Information Security, Policy & Records Office (ISPRO)

[www.virginia.edu/ispro](http://www.virginia.edu/ispro)

Email: IT-Security@Virginia.edu

**1E. The following procedures must also be followed.**

- Only investigators for this study and clinicians caring for the patient will have access to the data. They will each use a unique login ID and password that will keep confidential. The password should meet or exceed the standards described on the Information Technology Services (ITS) webpage about *[The Importance of Choosing Strong Passwords.](http://its.virginia.edu/accounts/passwords.html)*
- Each investigator will sign the [University’s Electronic Access Agreement](http://www.itc.virginia.edu/policy/form/eaa.pdf) forward the signed agreement to the appropriate department as instructed on the form.

If you currently have access to clinical data it is likely that you have already signed this form. You are not required to sign it again.

- UVa University Data Protection Standards will be followed

<http://www.virginia.edu/informationsecurity/dataprotection.>

- If identifiable data is transferred to any other location such as a desktop, laptop, memory stick, CD etc. the researcher must follow the University’s [“Electronic Storage of Highly Sensitive Data](https://policy.itc.virginia.edu/policy/policydisplay?id=IRM-015) Policy”. Additional requirements may be found in the Universities [Requirements for Securing Electronic Devices.](http://www.virginia.edu/informationsecurity/device-requirements.html)
- If identifiable health information is taken away from the UVa Health System, [Medical Center Policy # 0218](https://www.healthsystem.virginia.edu/documentation/manuals/mc/0218%20CLEAN%20Definition,%20Characteristics,%20Authentication%20and%20Maint.%20of%20the%20Medical%20Record%20and%20Designated%20Record%20Set.pdf?CFID=22015748&CFTOKEN=4a1e71196f4e4843-8C6A82B6) will be followed.
  - - - The data will be securely removed from the server, additional computer(s), and electronic media according to the University's [Electronic Data Removal Policy](https://etg07.itc.virginia.edu/policy/policydisplay?id=IRB-004).
      - The data will be encrypted or removed if the electronic device is sent outside of UVa for repair according to the University's [Electronic Data Removal Policy](https://etg07.itc.virginia.edu/policy/policydisplay?id=IRB-004).
      - If PHI will be faxed, researchers will follow the Health System Policy [# 0194.](https://www.healthsystem.virginia.edu/documentation/manuals/mc/0194FaxingofPatientInformation.pdf?CFID=22015748&CFTOKEN=4a1e71196f4e4843-8C6A82B6-082F-089F-715CE979D69D5497&jsessionid=8430dd77e667751342227e6469645c123183)
      - If PHI will be emailed, researchers will follow the Health System [Policy # 0193](http://www.healthsystem.virginia.edu/documentation/manuals/mc/0193ElectronicMail(E-mail).pdf?CFID=22015748&CFTOKEN=4a1e71196f4e4843-8C6A82B6-082F-089F-715CE979D69D5497&jsessionid=84308de86223c5f1e9f01d2a6f2059635c4d) and [University Data Protection Standards](https://www.virginia.edu/informationsecurity/dataprotection/documents/udps2-0.pdf) .
      - The data may not be analyzed for any other study without additional IRB approval.
      - If you are using patient information you must follow Health System [Policy # 0021](http://www.healthsystem.virginia.edu/documentation/manuals/mc/0021ConfidentialityofPatientInformation.pdf?CFID=22015748&CFTOKEN=4a1e71196f4e4843-8C6A82B6-082F-089F-715CE979D69D5497&jsessionid=84308de86223c5f1e9f01d2a6f2059635c4d).
      - Both data on paper and stored electronically will follow the [University's Record Management policy](https://policy.itc.virginia.edu/policy/policydisplay?id=IRM-017) and the Commonwealth statute regarding the Destruction of Public Records.

**Summary of Requirements to Comply with UVa Health System, Medical Center and University Policies and Guidance as noted above:**

**Highly Sensitive Data** is:

-personal information that can lead to identity theft if exposed or

-health information that reveals an individual’s health condition and/or history of health services use.

**Protected Health Information (PHI)** a type of Highly Sensitive Data, is health information combined with a HIPAA identifier

**Identifiable Health Information** under HIPAA regulations is considered to be *Highly Sensitive Data at UVa.*

A **Limited Data Set** (LDS) under HIPAA regulations is considered to be *Moderately Sensitive Data at UVa. The only HIPAA identifiers associated with data: dates and or postal address information limited to town or city, state, and zip code.* See Table A below for details.

| **Highly Sensitive Data (Identifiable Health Info per HIPAA )** | **Moderately Sensitive Data**  **(Limited Data Set and De-identified data per HIPAA)** |
| --- | --- |
| *General Issues* | *General Issues* |
| Discussions in private  Do not share with those not on the study team or those who do not have a need to know. | Do not share with those not on the study team or those who do not have a need to know |
| Password protect | Password protect |
| Physically secure (lock) hard copies at all times if not directly supervised.  If not supervised hard copies must have double protection (e.g. lock on room OR cabinet AND in building requiring swipe card for entrance). | Physically secure (lock) hard copies at all times if not directly supervised. |
| For electronic documents turn off File Sharing; turn on firewalls; use up to date antivirus and antispyware; delete data securely. | For electronic documents turn off File Sharing; turn on firewalls; use up to date antivirus and antispyware; delete data securely. |
| Encrypt  See [Encryption Solutions Guidance](http://www.virginia.edu/informationsecurity/encryption/)  *Files on Health System Network drives are automatically encrypted. If not stored there it is study teams responsibility to make sure data are encrypted.* |  |
| If device sent out for service or repair, encrypt or remove data AND contract for repair using a UVa Purchase order. | If device sent out for service or repair, encrypt or remove data AND contract for repair using a UVa Purchase order. |
| Store files on a network drive specifically designated for storing this type of data, e.g. high-level security servers managed by Information Technology Services or the “F” and “O” managed by Heath Systems Computing Services. You may access it via a shortcut icon on your desktop, but you are not allowed to take it off line to a local drive such as the desktop of your computer (e.g. C drive) or to an individual Use Device*. May access via VPN |  |
| Do not share with sponsor or other outside group before consent is obtained or the IRB has granted appropriate approvals and contract/ MTA is in place | Do not share with sponsor or other outside group before consent is obtained or the IRB has granted appropriate approvals and contract/ MTA is in place |
| If collected without consent/ HIPAA authorization will NOT be allowed to leave UVa HIPAA covered entity unless disclosure is approved by the IRB and the disclosure is tracked in EPIC | If collected without consent/ HIPAA authorization will NOT be allowed to leave UVa HIPAA covered entity unless disclosure is approved by the IRB and an MTA is in place prior to sharing of data |

| **Highly Sensitive Data (Identifiable Health Info per HIPAA )** | **Moderately Sensitive Data**  **(Limited Data Set and De-identified data per HIPAA)** |
| --- | --- |
| *Electronic Data Collection & Sharing* | *Electronic Data Collection & Sharing* |
| (e.g. smart phone app, electronic consent using tablet etc.)  MUST consult with ISPRO or Health System Web Development Office: 434-243-6702   - - - University Side: IT-Security@virginia.edu     - Health System: [Web Development Center:](http://www.healthsystem.virginia.edu/pub/web-development-center/web-development.html) |  |
| *Individual-Use Device* | *Individual‑Use Device* |
| Do not save to individual‑use device* without written approval of your Department AND VP  or Dean.  If approval obtained, data must be password  protected and encrypted. |  |
| Do not save an email attachment containing HSD to an individual use device  ( e.g. smart phone) |  |
| *E Mail* | *E Mail* |
| Do not share via email with Outlook Web/ or forward email using other email vendors like Gmail/ Yahoo |  |
| Do not send via email on smart phone unless phone is set up by Health System |  |
| Email may include name, medical record number or Social Security number only if sending email to or from a person with * HS in their email address.  *NOTE: VPR & IRB staff do not meet this criteria!* | In addition to sharing LDS, may include initials if persons sending and receiving email work within the UVa HIPAA covered entity.** |
| *FAX* | *FAX* |
| Verify FAX number before faxing | Verify FAX number before faxing |
| Use Fax Cover Sheet with Confidentiality Statement | Use Fax Cover Sheet with Confidentiality Statement |
| Verify receiving fax machine is in a restricted access area | Verify receiving fax machine is in a restricted access area |
| Verify intended recipient is clearly indicated | Verify intended recipient is clearly indicated |
| Recipient is alerted to the pending transmission and is available to pick it up immediately | Recipient is alerted to the pending transmission and is available to pick it up immediately |

| **Highly Sensitive Data (Identifiable Health Info per HIPAA )** | **Moderately Sensitive Data**  **(Limited Data Set and De-identified data per HIPAA)** |
| --- | --- |
| *Electronic Data Collection & Sharing* | *Electronic Data Collection & Sharing* |
| (e.g. smart phone app, electronic consent using tablet etc.)  MUST consult with ISPRO or Health System Web Development Office: 434-243-6702   - - - University Side: IT-Security@virginia.edu     - Health System: [Web Development Center:](http://www.healthsystem.virginia.edu/pub/web-development-center/web-development.html)   Contract must include required security measures. |  |
| May NOT be stored in places like UVaBox, UVaCollab, QuestionPro.  May also NOT be stored in non-UVa licensed cloud providers, such as Dropbox, Google Drive, SkyDrive, Survey Monkey, etc. | May be stored in places like UVaBox, UVaCollab, QuestionPro.  May NOT be stored in non-UVa licensed cloud providers, such as Dropbox, Google Drive, SkyDrive, Survey Monkey, etc. |
| *LOST OR STOLEN:* | *LOST OR STOLEN:* |
| Must report in accordance with protocol/ in accordance with the [Information Security Incident Reporting Policy](http://uvapolicy.virginia.edu/policy/IRM-012).  Any data breach will also be reported to the IRB of Record if the report meets the criteria of an Unanticipated Problem. | Must report in accordance with protocol/ in accordance with the [Information Security Incident Reporting Policy](http://uvapolicy.virginia.edu/policy/IRM-012).  Any data breach will also be reported to the IRB of Record if the report meets the criteria of an Unanticipated Problem. |

** Individual Use Device – examples include smart phone, CD, flash (thumb) drive, laptop, C drive of your computer.*

***The UVa HIPAA covered entity is composed of the UVa VP Office of Research, the Health System, School of Medicine, School of Nursing, Nutrition Services (Morrison’s), the Sheila C. Johnson Center, the Exercise and Sports Injury Laboratory and the Exercise Physiology Laboratory.*
